# Effect of SSRI exposure on the proliferation rate and glucose uptake in breast and ovary cancer cell lines

**DOI:** 10.1038/s41598-020-80850-9

**Published:** 2021-01-13

**Authors:** Britta Stapel, Catharina Melzer, Juliane von der Ohe, Peter Hillemanns, Stefan Bleich, Kai G. Kahl, Ralf Hass

**Affiliations:** 1 Department of Psychiatry, Social Psychiatry and Psychotherapy, Hannover Medical School, Carl-Neuberg Str. 1, 30625 Hannover, Germany; 2 Biochemistry and Tumor Biology Lab, Department of Gynaecology and Obstetrics, Hannover Medical School, Hannover, Germany

**Keywords:** Breast cancer, Ovarian cancer, Cell growth, Preclinical research, Depression

## Abstract

Breast cancer is the most prevalent malignancy amongst women worldwide while ovarian cancer represents the leading cause of death among gynecological malignancies. Women suffering from these cancers displayed heightened rates of major depressive disorder, and antidepressant treatment with selective serotonin reuptake inhibitors (SSRIs) is frequently recommended. Recently, narrative reviews and meta-analyses showed increased recurrence risks and mortality rates in SSRI-treated women with breast and ovarian cancer. We therefore examined whether three commonly prescribed SSRIs, fluoxetine, sertraline and citalopram, affect proliferation or glucose uptake of human breast and ovarian cancer cell lines characterized by different malignancies and metastatic potential. SSRI treatment or serotonin stimulation with therapeutically relevant concentrations over various time periods revealed no consistent dose- or time-dependent effect on proliferation rates. A marginal, but significant increase in glucose uptake was observed in SK-OV-3 ovarian cancer cells upon fluoxetine or sertraline, but not citalopram treatment. In three breast cancer cell lines and in two additional ovarian cancer cell lines no significant effect of SSRIs on glucose uptake was observed. Our data suggest that the observed increase in recurrence- and mortality rates in SSRI-treated cancer patients is unlikely to be linked to antidepressant therapies.

## Introduction

Major depression disorder (MDD) represents one of the preceding mood disorders worldwide with a 12-months prevalence of approximately 10% in the United States^1^. The World Health Organization predicted depression to be the leading cause of disease burden by 2030; it results in steadily increasing health care costs and a significant decline in quality of life. Compared to its already high prevalence in the general population, depression was found to be exceedingly more frequent in patients suffering from various physical affections, including oncologic diseases^2^. Hereby, the overall prevalence of depression found in cancer patients appears to depend on cancer type and the phase of treatment as well as the method used for diagnosis of depression^2^. In general, the common comorbidity of depression and oncologic diseases is related to a poor quality of life, it worsens the outcome of cancer patients, increases mortality and heightens medical costs^3,4^.

Breast cancer is the most prevalent malignancy amongst women worldwide while ovarian cancer represents the leading cause of death among gynecological malignancies^5^. Importantly, recent publications indicate that women suffering from malignant diseases display signs of anxiety and depression at the time of diagnosis with a higher frequency when compared to male cancer patients. Accordingly, patients diagnosed with gynecological malignancies are among the oncologic patients most likely to display depressive symptoms^6^.

Consequently, breast cancer patients were prescribed anti-depressants at the highest percentage when compared to any other cancer type^6^. Moreover, anti-depressant treatment appears also common in patients with ovarian cancer^7,8^.

Selective serotonin (5-HT) reuptake inhibitors (SSRIs) are the prevailing choice of medication in anti-depressive treatment in the general population as well as in cancer patients suffering from depression^9^. Clear guidelines regarding treatment of depression in patients with oncologic diseases are lacking; however, recent publications indicate pharmacologic intervention to be recommended for all cancer patients presenting with moderate or severe depression^10^. Additionally, SSRIs are a suggested treatment option for breast cancer patients to counteract side effects including hot flashes brought forward by anti-estrogen therapy for targeting of hormone-sensitive cancers^11^.

A considerable number of studies investigated the impact of SSRI treatment on cancer recurrence and survival in breast cancer patients, while research concerning effects of SSRIs on ovarian cancer progression appears less frequent. Results obtained vary significantly between studies that are oftentimes limited by small sample sizes and low number of events, especially when the effects of specific antidepressants are investigated. While a population-based retrospective cohort study by Chubak and colleagues found no increase in mortality but reported an augmented recurrence risk for patients that received paroxetine, a recent meta-analysis by Busby and colleagues reported an increase in mortality by 27% in breast cancer patients that received SSRIs for antidepressant treatment^12,13^. Interestingly, Busby et al. also reported that different SSRIs appeared to differentially affect the outcome of breast cancer patients and pointed out that the obtained results warranted further investigations concerning the effect of SSRIs in the context of breast cancer^13^. Concerning SSRI treatment of patients with epithelial ovarian cancer, a recent study found that SSRI use was associated with a significant decrease in time to disease progression while overall survival was not affected^14^.

SSRIs are thought to primarily act by inhibition of the 5-HT transporter (SERT) in pre-synaptic, serotonergic neurons, thereby decreasing 5-HT reuptake and increasing extracellular availability. However, different SSRIs were found to interact with alternate neurotransmitter receptors including but not limited to those of the serotonergic system (reviewed in^15–17^). These receptors were shown to be expressed also in different breast and ovarian cancer cell lines as well as tumor tissues^14,18–20^. Further, stimulating effects of 5-HT on breast and ovarian cancer cell survival, proliferation and metabolic activity were described^14,18,21,22^.

In line with the reported worse outcome of cancer patients receiving SSRI treatment, it was recently reported that SSRI treatment was associated with increased tumor cell proliferation rates in breast cancer tissues from late stage patients^23^. In this context, SSRIs amitriptyline and fluoxetine were found to potentiate tumor growth in a rat model of 7,12-Dimethylbenz[a]anthracene (DMBA)-induced mammary carcinogenesis^24^. Contrarily, some studies showed that drugs modifying 5-HT signaling, including SSRIs, inhibit tumor sphere formation in human breast tumor cells in in vitro and in vivo models, and fluoxetine was found to significantly decrease proliferation of several breast cancer cell lines by inducing apoptosis and autophagy-mediated cell death or endoplasmatic reticulum stress and autophagy, respectively^25–28^. While experimental research concerning the impact of SSRIs on ovarian cancer cells is less frequent when compared to studies analyzing breast cancer cells, fluoxetine was reported to induce apoptosis and decrease survival of ovarian tumor cells while contrarily, sertraline application resulted in a statistically not significant increase in tumor weight and in significantly more proliferating Ki67 positive cells within the tumor^14,29^.

The controversial clinical and experimental findings concerning SSRI-mediated effects on breast and ovary cancer cells and tumors warrant further studies especially as non-linear, dose-dependent effects of antidepressant drugs on cancer cell growth appear likely and might contribute to the observed discrepancies in cell culture and animal models^30^.

In the present study we demonstrate that the tested SSRIs, fluoxetine, sertraline and citalopram, which are frequently recommended for treatment of cancer-associated MDD, did not augment cell proliferation to a relevant level in various human breast and ovarian cancer cell lines and had only marginal or no impact on cellular glucose uptake.

## Results

### Marginal effects of low-dose, short-term SSRI stimulation on cell proliferation in human breast and ovarian cancer cell lines measured by Fluoroskan

**Figure 1:**
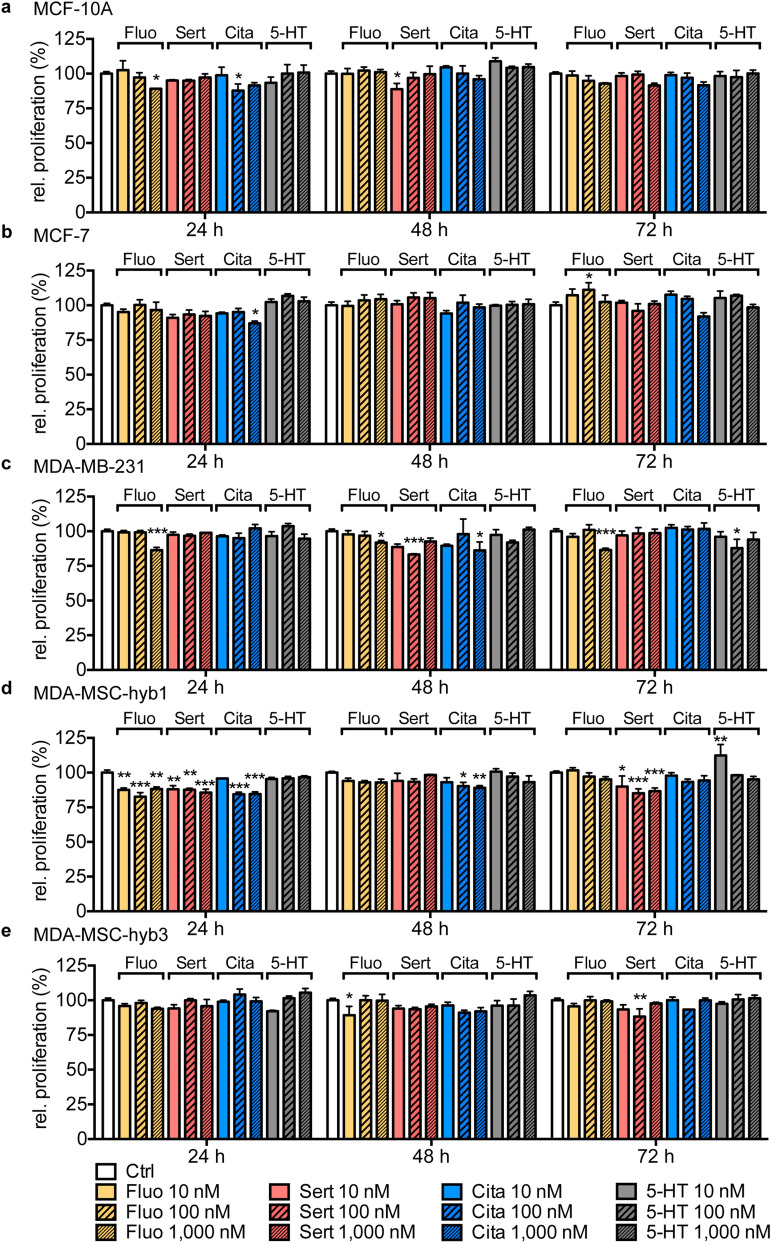
SSRI treatment or 5-HT exposure marginally impact cell proliferation of breast cancer cell lines. Bar graphs depict relative proliferation assessed by Fluoroskan of MCF-10A (**a**), MCF-7 (**b**), MDA-MB-231 (**c**), MDA-MSC-hyb1 (**d**) and MDA- MSC-hyb3 (**e**) cells in response to treatment with 10 nM, 100 nM or 1000 nM fluoxetine (Fluo; yellow), sertraline (Sert; red), citalopram (Cita; blue) or 5-HT (grey) for indicated time periods normalized to DMSO-treated control cells (Ctrl; white). Data are depicted as mean ± SEM and summarize n = 9–10 (Ctrl) and at least n = 3 (SSRI/5-HT) experiments. *P*-values were determined by two-way ANOVA followed by Dunnett’s multiple comparison test; ***P < 0.001; **P < 0.01; *P < 0.05 versus corresponding Ctrl.

**Figure 2:**
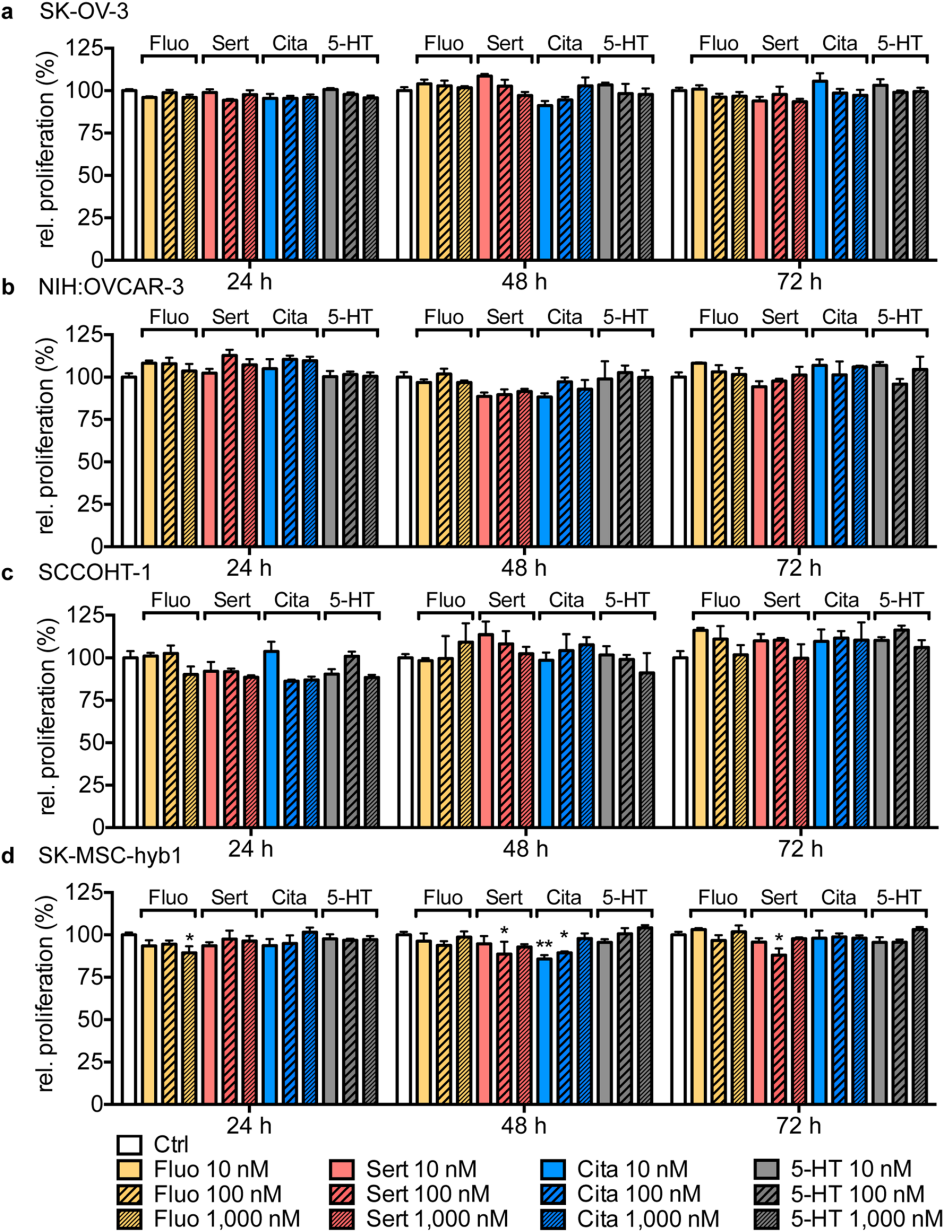
SSRI treatment and 5-HT stimulation have little effect on proliferation rates of ovarian cancer cell lines. Bar graphs depict relative proliferation measured by Fluoroskan of SK-OV-3 (**a**), NIH:OVCAR-3 (**b**), SCCOHT-1 (**c**) and SK-MSC-hyb1 (**d**) cells in response to exposure to 10 nM, 100 nM or 1000 nM fluoxetine (Fluo; yellow), sertraline (Sert; red), citalopram (Cita; blue) or 5-HT (grey) for indicated time periods normalized to DMSO-treated control cells (Ctrl; white). Data are depicted as mean ± SEM and summarize n = 10 (Ctrl) and n = 3 (SSRI/5-HT) experiments. *P*-values were determined by two-way ANOVA followed by Dunnett’s multiple comparison test, ***P < 0.001; **P < 0.01; *P < 0.05 versus corresponding Ctrl.

Proliferative effects of 5-HT were compared to three SSRIs (fluoxetine, citalopram, and sertraline) in five human neoplastic breast cancer (Fig. 1) and four ovarian carcinoma (Fig. 2) populations displaying different states of malignancy. The characteristics as well as the origin of the analyzed cell lines are summarized in suppl. Table S1. Incubation with low doses of 5-HT and the three SSRIs was performed at concentrations of 10 nM, 100 nM, and 1000 nM, which are in range of clinically relevant serum concentrations, for 24 h, 48 h, and 72 h, respectively^31^. Relative proliferation rates of investigated breast and ovarian cancer cell lines as analyzed by Fluoroskan assay, were only marginally affected by SSRI treatment or stimulation with 5-HT when compared to DMSO solvent controls. However, as inter-assay variances in the replication experiments are minimal, statistical significances were reached under distinct conditions in some cell lines. Suppl. Tables S2 and S3 summarize statistically significant drug concentrations and time point combinations and detail corresponding *P*-values for each cell line. In this regard, a dose- and time-dependent effect was only observed in MDA-MB-231 breast cancer cells that displayed a consistent decrease in cell proliferation upon treatment with the highest concentration of fluoxetine at all three analyzed time points (Fig. 1c). Otherwise, no dose- or time-dependent effects were observed in all other analyzed cell lines and punctual changes represent mostly small decreases in proliferation rates (Figs. 1 and 2). Importantly, treatment of carcinoma cells with higher concentrations of the chemotherapeutic compound carboplatin used as an internal control for the Fluoroskan assay resulted in certain dose-dependent inhibitory effects on cell proliferation although not directly comparable to SSRI treatment, (suppl. Figs. S1 and S2) as described previously^32–34^.

### Measurement of cell viability by MTT assay confirms minimal effects of low-dose, short-term SSRI treatment on human breast and ovarian cell lines

To confirm the results obtained by Fluoroskan assays, we utilized the MTT (3-[4,5-dimethylthiazol-2-yl]-2,5 diphenyl tetrazolium bromide) assay as a secondary, independent method to determine viable cells in response to low dose SSRI- and 5-HT treatment with concentrations of 100 and 1000 nM. As the MTT assay is based on the mitochondrial conversion of MTT to formazan crystals, it is considered an indicator for cell viability oftentimes used in the context of in vitro cytotoxicity studies, as total mitochondrial activity is related to the number of viable cells in most cell lines^35^. In agreement with the data obtained by Fluoroskan assay, short-term (24–72 h) SSRI- or 5-HT treatment did not result in dose- or time-dependent changes of the number of viable cells in any of the analyzed breast (suppl. Fig. S3) or ovarian cell lines (suppl. Fig. S4). As for the results obtained by Fluoroskan assay, some punctual, statistically significant changes in relative absorbance were reached for some cell lines that are summarized including corresponding *P*-values in suppl. Tables S4 and S5. Overall, results of the MTT assays confirmed the findings obtained by Fluoroskan assay. In particular, low doses of SSRIs that are within the physiological, therapeutic range elicit only marginal effects on human breast and ovarian cancer cell proliferation and viability.

### Stimulation with high fluoxetine concentrations does not impact cell cycle traverse

**Figure 3:**
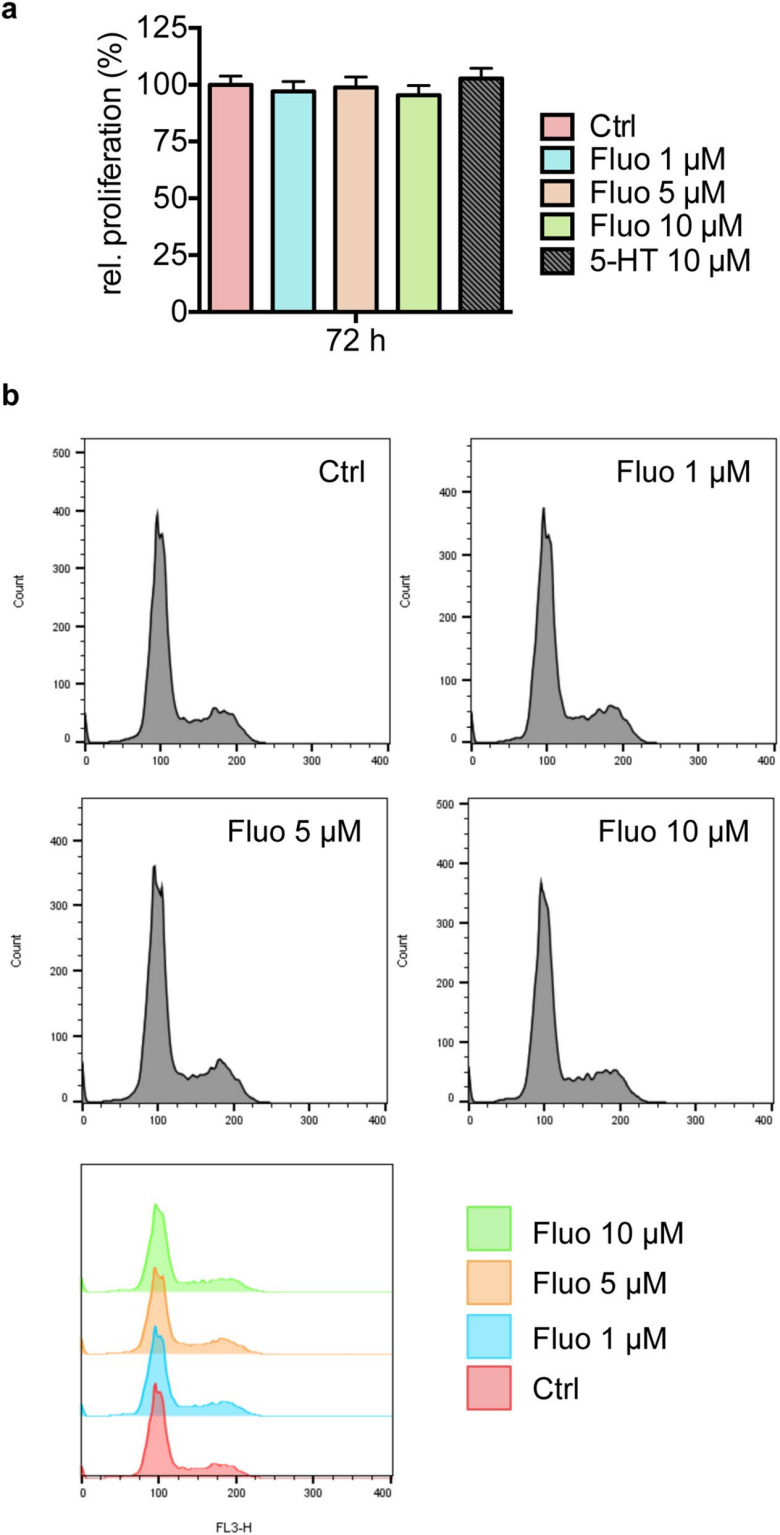
Higher concentrations of fluoxetine have no significant impact on proliferation and cell cycle progression of MDA-MB-231 breast cancer cells. (**a**) Bar graph depicts relative proliferation of MDA-MB-231 breast cancer cells in response to 72 h stimulation with 1 µM, 5 µM or 10 µM fluoxetine (Fluo) compared to treatment with 10 µM 5-HT or corresponding DMSO-treated control cells (Ctrl) as assessed by Fluoroskan. (**b**) Histograms depict representative results of cell cycle analysis of MDA-MB-231 cells in response to fluoxetine (Fluo) stimulation with indicated concentrations for 72 h. Data in (**a**) derive from n = 10 experiments and are depicted as means ± SEM. *P*-values were computed by one-way ANOVA followed by Dunnett’s multiple comparison test.

As we observed a consistent small but significant decrease in proliferation of MDA-MB-231 breast cancer cells at the highest fluoxetine concentration of 1 µM at all three analyzed time points in Fluoroskan assays that was not detected by MTT assay, we investigated the potential effect of higher fluoxetine concentrations at 1 µM, 5 µM, and 10 µM as compared to 10 µM 5-HT in this cell line. Incubation of MDA-MB-231 breast cancer cells for 72 h with indicated concentrations of fluoxetine demonstrated no significant differences in the proliferation rates (Fig. 3a).

Likewise, no differences were observed in the cell cycle traverse of MDA-MB-231 breast cancer cells or in response to treatment with fluoxetine (1 µM, 5 µM, and 10 µM) for 72 h when compared to corresponding DMSO-treated control cells (Fig. 3b).

### Prolonged SSRI stimulation up to 144 h does not consistently impact viability of human breast and ovarian cancer cell lines as assessed by MTT assay

**Figure 4:**
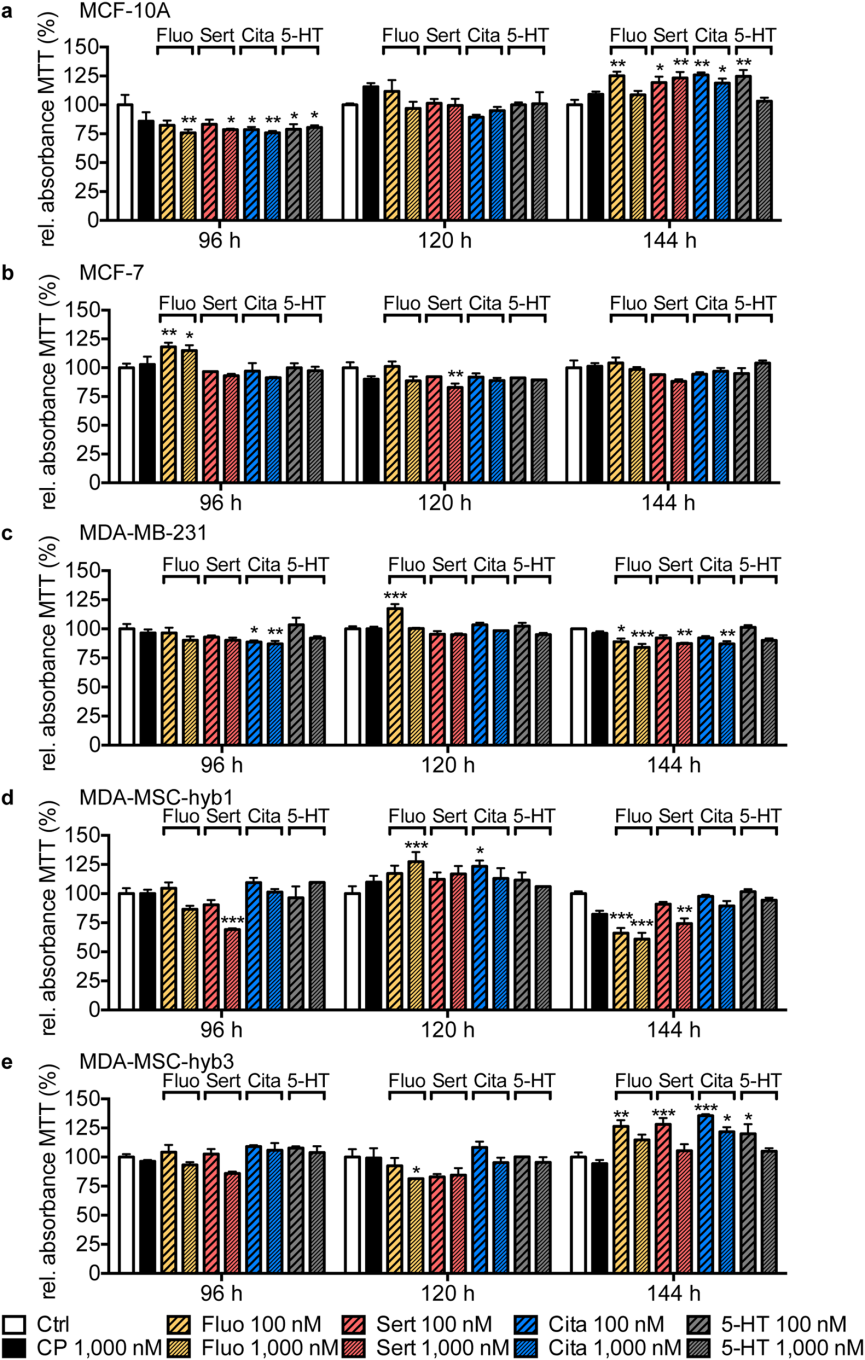
Effect of low-dose, long-term SSRI treatment or 5-HT exposure on breast cancer cell lines measured by MTT assay. Bar graphs depict relative absorbance of MTT assay of MCF-10A (**a**), MCF-7 (**b**), MDA-MB-231 (**c**), MDA-MSC-hyb1 (**d**) and MDA-MSC-hyb3 (**e**) cells in response to treatment with 100 nM or 1000 nM fluoxetine (Fluo; yellow), sertraline (Sert; red), citalopram (Cita; blue) or 5-HT (grey) for indicated time periods (96 h to 144 h) compared to DMSO-treated control cells (Ctrl; white) and cells receiving carboplatin (CP; 1000 nM; black). Data are depicted as mean ± SEM and summarize n = 3 experiments. *P*-values were determined by two-way ANOVA followed by Dunnett’s multiple comparison test; ***P < 0.001; **P < 0.01; *P < 0.05 versus corresponding Ctrl.

**Figure 5:**
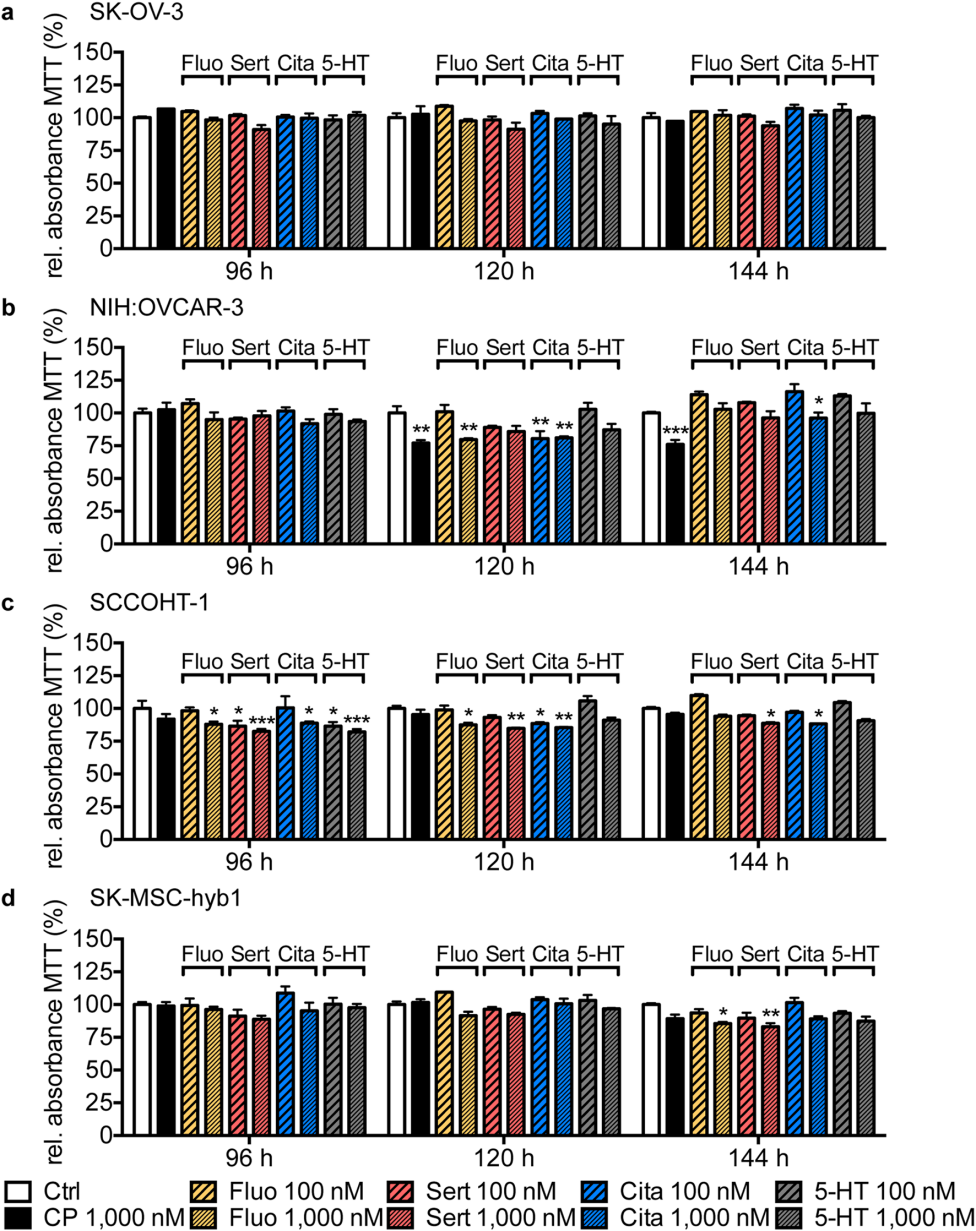
Effect of low-dose, long-term SSRI treatment or 5-HT exposure on ovarian cancer cell lines measured by MTT assay. Bar graphs depict relative absorbance of MTT assay of SK-OV-3 (**a**), NIH:OVCAR-3 (**b**), SCCOHT-1 (**c**) and SK-MSC-hyb1 (**d**) cells in response to exposure to 100 nM or 1,000 nM fluoxetine (Fluo; yellow), sertraline (Sert; red), citalopram (Cita; blue) or 5-HT (grey) for indicated time periods (96 h to 144 h) compared to DMSO-treated control cells (Ctrl; white) and cell receiving carboplatin (CP; 1,000 nM; black). Data are depicted as mean ± SEM and summarize n = 3 experiments. *P*-values were determined by two-way ANOVA followed by Dunnett’s multiple comparison-test; ***P < 0.001; **P < 0.01; *P < 0.05 versus corresponding Ctrl.

To exclude the possibility of long-term effects of low-dose of SSRIs treatment, we utilized the MTT method to assess cell viability of human breast (Fig. 4) and ovarian cancer cell lines (Fig. 5) in response to fluoxetine, sertraline, citalopram or 5-HT at concentrations of 100 nM or 1000 nM in comparison to corresponding control cells or cells that were treated with carboplatin (1000 nM) for 96 h, 120 h, or 144 h. Similar to short-term treatments, no consistent dose- or time-dependent effects were detectable in the analyzed cell lines for most of the tested SSRIs. Punctual, statistically significant changes in cell viability measured as relative absorbance of MTT and corresponding *P*-values are summarized in suppl. Tables S5 and S6. As the higher sertraline concentration of 1000 nM evoked a small but statistically significant decrease in the MTT signal in SCCOHT-1 cells at all analyzed time points, we additionally measured proliferation rate of SCCOHT-1 cells by Fluoroskan assay. Different to the results of the MTT assay, no significant changes in cell proliferation of SCCOHT-1 cells was observed in response the stimulation with 1000 nM sertraline in any of the analyzed long-term time points (suppl. Fig. S5).

### High concentrations of SSRIs decrease viable cell number measured by MTT assay in most analyzed breast and ovarian cancer cell lines

Since previous studies reported cell toxic rather than proliferation-increasing effects upon stimulation with higher SSRI concentrations, we assessed viable cell numbers of breast and ovarian cancer cell lines in response to SSRI or 5-HT exposure at concentrations of 10 µM and 100 µM using MTT assay. Here, we observed dose- as well as time dependent effects in most of the analyzed cell lines.

As expected, at a concentration of 100 µM the tested SSRIs significantly decreased relative MTT absorbance in all analyzed breast and ovarian cancer cell lines within 72 h except for SCCOHT-1 cells (suppl. Figs. S6 and S7). This effect persisted up to 144 h (suppl. Figs. S8 and S9). In SCCOHT-1 cells significant decreases in viable cell counts were less pronounced indicating a decreased susceptibility to SSRI-induced cell toxicity.

In contrast, treatment with SSRIs at a concentration of 10 µM demonstrated differences between the tested SSRIs. Sertraline, but not fluoxetine or citalopram, significantly decreased viable cell count in most of the analyzed cell lines (with the exception of MDA-MB-231 and SCCOHT-1 cells) within the first 72 h of treatment (suppl. Figs. S5 and S6). Longer treatment periods resulted in decreased cell viability in response to fluoxetine exposure, while citalopram did not elicit consistent significant effects on cell viability at a concentration of 10 µM (suppl. Figs. S8 and S9). In contrast, stimulation with 5-HT did not markedly decrease viability in any of the analyzed cell lines. However, stimulation of SCCOHT-1 cells with 10 µM but not 100 µM significantly increased viable cell counts up to the level of the 72 h time point (suppl. Fig. S7).

### Glucose uptake is only marginally affected by SSRI stimulation

**Figure 6:**
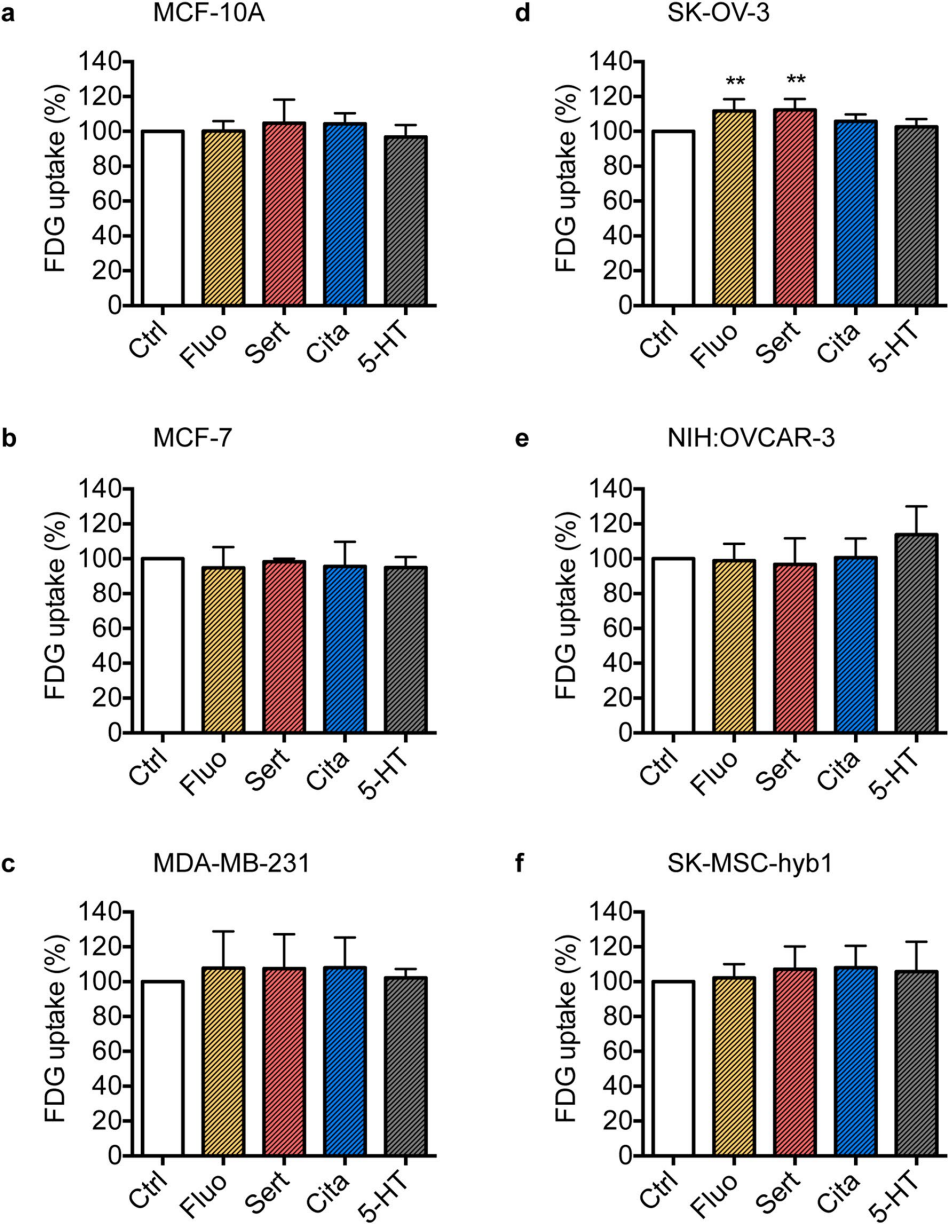
Fluoxetine and sertraline marginally increase glucose uptake in SK-OV-3 cells. Bar graphs depict relative cellular glucose uptake assessed by 18F-FDG incorporation of MCF-10A (**a**), MCF-7 (**b**) and MDA-MB-231 (**c**) breast cancer as well as SK-OV-3 (**d**) NIH:OVCAR-3 (**e**) and SK-MSC-hyb1 (**f**) ovarian cancer cells in response to stimulation with 1 µM fluoxetine (Fluo), sertraline (Sert), citalopram (Cita), or 5-HT relative to DMSO-treated control cells (Ctrl) for 72 h. Data, derived from n = 3 (b and e), n = 4 (a and c) or n = 5 (d and e) independent cell passages performed in duplicates, are depicted as means ± SEM. *P*-values were computed by one-way ANOVA followed by Dunnett’s multiple comparison test; **P < 0.01.

As proliferation rates of cancer cells essentially depend on adequate substrate availability and have been shown to be increased in the presents of high concentrations of the main energy substrate glucose^36^, we investigated glucose uptake of three breast (MCF-10A, MCF-7, MDA-MB-231) and three ovarian carcinoma (SK-OV3, NIH:OVCAR-3, SK-MSC-hyb1) cell lines by use of the radionuclide-labeled glucose analog (18)F-fluorodeoxyglucose (18F-FDG) in response to SSRI treatment. Stimulation with 1000 nM fluoxetine or sertraline but not citalopram resulted in marginal but significant increases in cellular glucose uptake in SK-OV-3 cells (Fluo vs. Ctrl: *P* = 0.0041, Sert vs. Ctrl: *P* = 0.0027) while no significant effects of SSRI- or 5-HT stimulation were observed in any of the other investigated cell lines upon 72 h of stimulation when compared to DMSO-treated control cells (Fig. 6).

## Discussion

While guidelines brought forward and agreed to by the US, the EU and Japan regulate testing for genotoxicity and carcinogenicity for all pharmaceuticals that are used for at least 6 month or frequently in an intermitted manner, a review summarizing genotoxic and carcinogenic effects of routinely used antipsychotics and antidepressants found that only 8 antipsychotics and 8 antidepressants out of the 104 drugs reviewed were tested in substantial agreement to these guidelines (reviewed in^37^). With regards to these findings and factoring in recent publications describing adverse outcomes of breast and ovarian cancer patients receiving SSRI therapies, a better understanding of antidepressant side effects on cancer cells is inevitable especially considering that the aforementioned guidelines do not include recommendations concerning potential SSRI-mediated effects on already established tumors.

Although limited to in vitro settings, low doses up to 1000 nM within the reported therapeutic range were associated with no relevant increases in proliferation rate that manifested in a dose- or time-dependent manner in any of the analyzed human breast and ovarian cancer cell lines. While initial screening experiments hinted towards a marginally decreased proliferation rate of MDA-MB-231 breast cancer cells upon stimulation with a high concentration of fluoxetine (1000 nM) at all analyzed time points, further experiments with high fluoxetine concentrations for 72 h did not confirm inhibitory effects on proliferation in this cell line and additional cell cycle analyses showed no differences between fluoxetine stimulated cells when compared to corresponding MDA-MB-231 control cells. Similar findings were observed in response to treatment with 1000 nM sertraline in SCCOHT-1 cells.

A direct or indirect (5-HT mediated) effect of SSRIs on cancer cells appears likely, as in addition to their inhibitory action on the SERT, interaction of different SSRIs with alternate neurotransmitter receptors that are expressed on breast and ovarian cancer tissues and cell lines was described (reviewed in^15–17^)^14,18–20^. Furthermore, as platelets that represent one of the major storage compartment for 5-HT in the blood, express the brain 5-HT transporter SERT, it appears reasonable that SSRIs might not only affect brain but also blood extracellular 5-HT concentrations as well as 5-HT levels in the microenvironment of tumors. In this regard, a study with a limited number of patients found that fluoxetine treatment resulted in decreased 5-HT serum concentrations and a complete knock out of the SERT gene similarly resulted in decreased 5-HT serum content in a transgenic mouse model^38,39^. Previous cell culture studies have described increased proliferation of MDA-MB-231 cells in response to stimulation with 5-HT for 72 h^19^. In agreement with these findings by Gautman et al. 5-HT stimulation of MDA-MB-231 and MCF-7 cells at serum-starved (0.2% FCS) conditions resulted in a significant increase in relative cell count measured by MTT assay (suppl. Fig. S10). Importantly, cell proliferation was significantly hampered in serum-starved control cells and even decreased in the MDA-MB-231 cell line (suppl. Fig. S10a,b) indicating suboptimal proliferative conditions. Moreover, the findings suggested different 5-HT effects on cell growth during starvation-mediated cell stress as compared to an optimized culture, which resulted in only minor effects of 5-HT stimulation. These results are in line with previous studies showing differential effects of 5-HT on breast cancer cells in the presence of different FCS concentrations^18^.

Experimental studies concerning SSRI-mediated effects on breast or ovary cancer cell proliferation and survival in vitro and cancer progression in vivo yielded controversial results. However, oftentimes experimental studies reported beneficial effects of SSRIs and other 5-HT modulating drugs, as these compounds appear to decrease breast and ovarian cancer cell proliferation and survival, which is in contrast to clinical data^25–28^. These discrepancies concerning clinical observations and experimental studies might potentially be attributed to differences in drug concentrations in experimental settings when compared to those reached in the human circulation and tumor microenvironment. In this regard, several studies investigating SSRI-mediated effects on cell proliferation and survival in cell culture systems, used concentrations ranging from 2.5 to 15 µM, which considerably exceed therapeutic drug levels in serum of patients^25,26,29–31^.

The primary focus of our study was the analysis of effects brought forward by SSRI concentrations within the therapeutic range commonly found in plasma of patients receiving SSRIs for treatment of MDD. Furthermore, we analyzed several time points up to 144 h to account for potential time-dependent effects and performed the analyses in five individual breast- and four ovarian cancer cell lines differing in malignancy and metastatic potential to account for potential cell line specific effects.

The importance to investigate effects of lower drug concentrations within the nanomolar range is highlighted by publications indicating potential non-linear, dose-dependent effects which might lead to opposite effects compared to the higher concentrations used in previous studies^30^.

However, to ensure comparability of our experimental set up with previous studies, we included a set of experiments utilizing higher drug concentrations.

In this regard, our results are in line with a study by Bowie and colleagues that found significant effects on cell viability of various human breast cancer cell lines including MCF-10A cells only at fluoxetine concentration exceeding 5 µM while lower concentrations had no significant impact over stimulation periods from 24 to 76 h^28^. Similarly, we observed a significant decrease in viable cell counts in response to treatment of MCF-10A cells with 10 µM fluoxetine. However, even at fluoxetine concentrations up to 10 µM we did not observe significant changes in proliferation rate in MDA-MB-231 breast cancer cells. A corresponding cell cycle analysis revealed no significant changes in comparison to DMSO-treated control cells of this cell line indicating varying susceptibility to cytotoxic effects of SSRIs between different human breast and ovarian cancer cell lines. A potential explanation for these discrepancies to previous results by others may include different experimental set ups and culture conditions. Importantly, we observed significant differences in the cytotoxic potential of the analyzed SSRIs with sertraline eliciting adverse effects on viable cell counts at a concentration of 10 µM within the first 72 h of treatment. Conversely, fluoxetine treatment at the same concentration resulted in decreased cell viability in longer treatment periods of more that 96 h in most cell lines. Of interest, citalopram treatment displayed little if any influence on the cell viability at a concentration of 10 µM. Importantly, based on meta-analysis reporting increased cancer recurrence and mortality in cancer patients receiving SSRIs^13,14^, neither of the tested SSRIs directly elevated cell viability in any of the analyzed cell lines.

An increase in glucose oxidation via glycolysis is a hallmark of cancer cells when compared to non-cancerous cells and tissues and increased glucose concentrations were associated with accelerated cell proliferation rates in endometric as well as in certain breast cancer cells in vitro and in vivo^36,40–42^. Additionally, various clinical conditions that are characterized by hyperglycemia, including next to others chronic stress and cancer itself, were found to be associated with increased tumorigenesis and tumor progression^43,44^. The importance of glucose metabolism for cancer development and progression is furthermore highlighted when considering that tumor diagnostics (in form of FDG-PET) as well as newer tumor therapeutics target the high glucose uptake and oxidation of cancer cells^45–47^. As glucose uptake across the cell membrane is considered a rate-limiting step in the metabolism of glucose^48^ and studies analyzing direct effects of SSRIs on cellular glucose uptake in breast and ovarian cancer cell lines are lacking, we subsequently assessed cellular glucose uptake in response to SSRI stimulation. Albeit we increased SSRI concentrations to 1 µM, we only observed a marginal but statistically significant increase in glucose uptake upon fluoxetine and sertraline but not citalopram stimulation in SK-OV-3 ovarian cancer cells. While this increase was not associated with an increase of cell proliferation at the same experimental time point and drug concentrations, it warrants further investigations, as previous studies reported not only an association of glucose metabolism and cancer cell proliferation, but also a link between metabolic flexibility and metastatic processes^49^. Considering that we did not observe an SSRI-mediated effect on any of the other analyzed cell lines, it appears likely that certain SSRIs influence glucose uptake in a cell type specific manner.

MDD poses an additional burden on patients suffering form breast- or ovarian cancer. Beyond worsening subjective lifestyle, drug adherence and quality of life, MDD also increases the risk for suicide in cancer patients^50^. Therefore, identification and treatment of MDD is an important step in the multimodal treatment plan for cancer patients. Considering our results, it appears unlikely that the observed worsened outcome of breast and ovarian cancer patients receiving SSRIs for treatment of MDD is brought forward by direct effects of the examined SSRIs fluoxetine, sertraline, or citalopram, or indirectly by modulation of peripheral 5-HT concentrations.

The choice of an antidepressant drug is a challenging task, since side effects, pharmacologic interactions and safety aspects concerning cancer progression have to be taken into consideration. Given that our data so far do not support the hypothesis that SSRIs per se may be responsible for the observed negative effects on mortality in antidepressant treated depressed cancer patients, the important benefits of SSRI should be considered. Further studies are warranted to systematically examine other frequently used drugs in comorbid breast- and ovarian cancer patients (i.e. anxiolytics, hypnotics, mood stabilizers and other antidepressant agents).

## Conclusion

Together, our data demonstrate little if any interference of the tested SSRIs at low concentrations with the proliferative capacity and glucose uptake of the investigated cancer cells. Additionally, our data confirm previous studies reporting cell toxic effects of high-dose SSRI exposure on cancer cells. Although limited to in vitro results, treatment of MDD with the tested SSRIs may support breast or ovarian cancer patients by improving psychopathology and quality of life.

### Limitations

Our study is limited to in vitro experiments in which cancer cells are provided with optimal growth conditions and interaction with other cell types cannot be accounted for. Furthermore, we did not study the effect of SSRIs in the context of other cancer medications, which might be of importance considering the established inhibitory effect that especially fluoxetine exhibits on cytochrome P450 enzymes^51–53^, which could affect effectiveness of anti-cancer medications metabolized via this system^54^.

While we choose SSRI concentrations that cover the therapeutic range normally found in patients being treated for MDD, we can only assume that similar concentrations of antidepressants are also present in the tumor microenvironment, as we are unaware of studies that investigated SSRI concentrations within tumors.

## Material and methods

### Breast- and ovarian cancer cell lines

The human SK-OV-3 ovarian carcinoma cell line and human breast carcinoma cell lines MCF-10A, MCF-7 and MDA-MB-231 were obtained from ATCC. The human NIH:OVCAR-3 ovarian cancer cell line was purchased from I.A.Z., Munich, Germany. Highly malignant and metastasizing human MDA-MSC-hyb1 breast cancer cells and lower malignant human MDA-MSC-hyb3 breast cancer cells were isolated from cancer cell fusion of MDA-MB-231 cells with mesenchymal stroma/stem-like cells as described^55,56^. Likewise, SK-MSC-hyb1 represents a human ovarian cancer fusion population of SK-OV-3 cells with mesenchymal stroma/stem-like cells^57^. SCCOHT-1 cells represent a rare form of small cell hypercalcemic ovarian cancer^58^. Cancer cell lines and their origin and characteristics are also summarized in suppl. Table S1.

### Cell culture conditions

The benign breast cancer cell line MCF-10A was grown in phenol red-free Mammary Epithelial Cell Basal Medium (MECBM) with appropriate supplement mix (PromoCell, Heidelberg, Germany). MCF-7 breast cancer cells were cultivated in Dulbecco’s modified Eagle’s medium supplemented with either 0.2% (v/v) or 10% (v/v) fetal calf serum (FCS), 100 U/mL penicillin, 100 µg/mL streptomycin and 2 mM l-glutamine. The triple-negative breast cancer cell line MDA-MB-231 was cultured in Leibovitz’s l-15-medium supplemented with 10% (v/v) FCS, 100 U/mL penicillin, 100 µg/mL streptomycin and 2 mM l-glutamine^59^. MDA- MSC-hyb1, MDA- MSC-hyb3, and SK-MSC-hyb1 cancer fusion cells^60,61^ were cultured in αMEM supplemented with 10% allogenic human AB-serum (blood from 31 male AB donors was commercially obtained from a blood bank, Hannover Medical School, Germany, and processed to serum), 100 U/mL penicillin, 100 µg/mL streptomycin and 2 mM l-glutamine. The ovarian cancer populations SK-OV-3, NIH:OVCAR-3, and SCCOHT-1 were cultivated in RPMI 1640 supplemented with 10% (v/v) FCS, 2 mM l-glutamine, 100 U/mL penicillin and 100 µg/mL streptomycin^57^.

Authentication of the cell lines was performed by short tandem repeat (STR) fragment analysis using the GenomeLab human STR primer set (Beckman Coulter Inc., Fullerton, CA, USA) as previously described^62^. Cells were tested for mycoplasma by the luminometric MycoAlert Plus mycoplasma detection kit (Lonza Inc., Rockland, ME, USA) according to the manufacturer’s recommendations.

### Measurement of cell proliferation by Fluoroskan

Proliferation of cells was analyzed by Fluoroskan quantification following fluorescence labeling by stable transduction of the cell lines with a third generation lentiviral SIN vector carrying either the eGFP gene or the mCherry gene^63^. Stimulation of cells was carried out in culture medium as detailed above containing indicated serum concentrations to ensure optimal steady-state conditions for cell growth. Labeled cells (1,000 cells/well for short-term (24–72 h) and 500 cells/well for long-term (96–144 h)) were incubated in flat bottom 96-well plates in the absence (controls) or in the presence of the SSRIs fluoxetine, sertraline, and citalopram (Selleckchem) at clinically relevant concentrations (10–1000 nM) as well as with 5-HT (Selleckchem). At indicated time points, the medium was removed and cells were lysed with 10% SDS following detection of fluorescence intensity of mCherry (excitation 485 nm, emission 612 nm) and eGFP (excitation 485 nm, emission 520 nm) using the Fluoroskan Ascent FL (Thermo Fisher Scientific, Schwerte, Germany) since the fluorescence intensity is proportional to the cell number^59^. Relative proliferation rates were calculated as percentage of controls.

### Measurement of cell viability by MTT assay

The colorimetric MTT (3-(4,5-dimethylthiazol-2-yl)-2,5-diphenyltetrazolium bromide) assay is based on the reduction of the yellow tetrazolium salt by metabolically active cells to generate purple formazan crystals. Following drug incubation (100 nM–100 µM, as indicated in the respective result sections), the different cell lines were incubated with 1 mg/mL MTT (Sigma) for 3 h. Thereafter, the reaction was stopped by addition of DMSO and measured at 540 nm using the Multiskan Ex Elisa plate reader (ThermoFisher). Results are depicted as relative absorbance in comparison to control cells.

### Cell cycle analysis

Analysis and quantification of the different cell cycle phases was performed as described elsewhere^64^. Briefly, 10^5^ cells were fixed in 70% (v/v) ice-cold ethanol at 4 °C for 24 h. Thereafter, fixed cells were stained with propidium-iodide for 30 min at room temperature. The samples were subsequently measured in a FACSCalibur (BD Biosciences, Singapore) flow cytometer and analyzed using the FlowJo V10 software.

### Measurement of cellular glucose uptake by (18)F-fluorodeoxyglucose incorporation

Cellular glucose uptake in breast cancer (MCF-10A, MCF-7 and MDA-MB-231) and ovarian cancer (SK-OV-3, NIH:OVCAR3 and SK-MSC-hyb1) cell lines was assessed by incorporation of the radionuclide-labeled glucose analog 18F-FDG. Cells were cultured in the presence of fluoxetine, sertraline, citalopram, and 5-HT at a concentration of 1 µM for 72 h in a 6-well format and suitable culture medium as outlined above. Before measurement, cells were washed twice with glucose-free RPMI medium followed by 30 min of incubation under glucose-free conditions at 37 °C. To each well 250 kBq 18F-FDG were added and cells were incubated for additional 30 min at 37 °C. Cells were washed twice with glucose-containing medium followed by cell lysis in RIPA buffer containing protease inhibitors. Radioactivity in cell lysates was measured by use of an automated gamma counter as counts per minute (CPM). To account for decay, values were corrected to the start of measurement. Obtained values were normalized to protein content assessed by the Bradford method in accordance to the manufacture’s instructions (BioRad, Munich, Germany).

### Statistical analyses

GraphPad Prism 6 software was used to compute all statistical analyses. One-way ANOVA or two-way ANOVA followed by Dunnett’s multiple comparison test or Sidak’s multiple comparison test, were utilized as applicable and indicated in the respective figure legends. Individual statistical tests and number of experimental repeats are included in the respective figure legends. Differences were considered to be statistically significant for *P*-values < 0.05 and data are depicted as means ± SEM throughout the manuscript. Control cells were set as 100% in all experiments.

## Supplementary Information

Supplementary Information
