# Supplementary material for: Effect of SSRI exposure on the proliferation rate and glucose uptake in breast and ovary cancer cell lines
Source: Sci Rep. 2021 Jan 13;11:1250. doi: 10.1038/s41598-020-80850-9 (PMC7806821; doi:10.1038/s41598-020-80850-9)
Supplement: Supplementary file 1 — Supplementary Information [file 41598_2020_80850_MOESM1_ESM.pdf]

Effect of SSRI exposure on the proliferation rate and glucose uptake in breast and ovary cancer cell lines

Authors: Britta Stapel[1]*, Catharina Melzer[2], Juliane von der Ohe[2], Peter Hillemanns[2], Stefan Bleich[1], Kai G. Kahl[1], Ralf Hass[2]

Authors affiliations:

1) Department of Psychiatry, Social Psychiatry and Psychotherapy, Hannover Medical School

2) Biochemistry and Tumor Biology Lab, Department of Gynaecology and Obstetrics, Hannover Medical School

Supplemental Information

Supplemental Information

Supplemental Table S1: Characteristics of human breast- and ovarian cancer cell lines

Cell line	Origin	Characteristics
MCF-10A [1]	human, mammary gland, breast, fibrocystic disease, epithelial	ER-negative, non-invasive
MCF-7 [2]	human, mammary gland, breast, metastatic site, pleural effusion, epithelial	ER-positive, poorly-aggressive, low metastatic potential
MDA-MB-231 [3]	human, mammary gland, breast, metastatic site, pleural effusion, epithelial	triple negative (ER-, PR-, Her2-negative), aggressive tumor growth and metastases
MDA-MSC-hyb1 [4]	human, derived from fusion of MDA-MB-231 with mesenchymal stroma/stem-like cells	high malignancy and metastatic potential
MDA-MSC-hyb3 [5]	human, derived from fusion of MDA-MB-231 with mesenchymal stroma/stem-like cells	low malignancy and metastatic potential
SK-OV-3 [6]	human, ovary, epithelial derived ascites of an ovarian serous cystadenocarcinoma	prominent subcutaneous tumor development in NOD SCID mice
NIH:OVCAR-3 [7, 8]	human, ovary, epithelial, derived from ascites of a progressive adenocarcinoma of the ovary	distinct chemotherapeutic sensitivity as compared to SK-OV-3 or SCCOHT-1 cells, hormone receptors
SCCOHT-1 [9]	human, derived from biopsy of ovarian small-cell carcinoma of the hypercalcemic type, FIGO Ia	cell culture model for SCCOHT, expression of CD90 and vimentin as mesenchymal-like markers and NCAM/CD56 as neuroendocrine marker
SK-MSC-hyb1 [10]	human, derived from fusion of SK-OV-3 with mesenchymal stroma/stem-like cells	diminished proliferative capacity and reduced malignancy as compared to the parental SK-OV-3 cells

CD: cluster of differentiation, ER: estrogen receptor, FIGO: International Federation of Gynecology and Obstetrics, Her2: human epidermal growth factor receptor 2, NCAM: neural cell adhesion molecule, PR: progesterone receptor, SCCOHT: small cell carcinoma of the ovary hypercalcemic type.

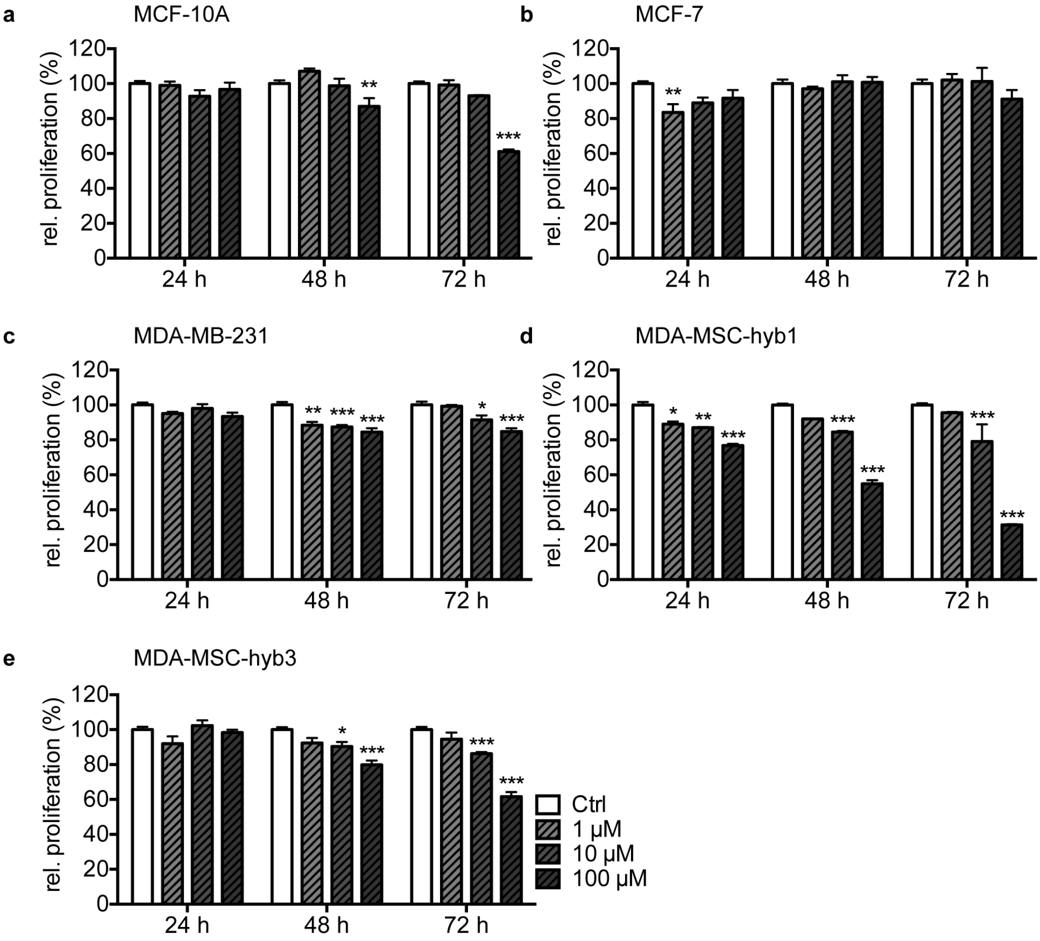

Supplemental Figure S1: Effect of carboplatin treatment on cell proliferation of breast cancer cell lines. Bar graphs depict relative proliferation rates of MCF-10A (**a**), MCF-7 (**b**), MDA-MB-231 (**c**), MDA-MSC-hyb1 (**d**) and MDA-MSC-hyb3 (**e**) cells in response to treatment with 1 µM, 10 µM or 100 µM carboplatin in comparison to DMSO-treated control cells (Ctrl) for indicated time points. Data derive from n = 9-10 (Ctrl) or n = 3 (carboplatin) experiments and are depicted as means ± SEM. *P*-values were computed by two-way ANOVA followed by Dunnett's multiple comparison test; ***$P < 0.001$; **$P < 0.01$; *$P < 0.05$ versus corresponding Ctrl.

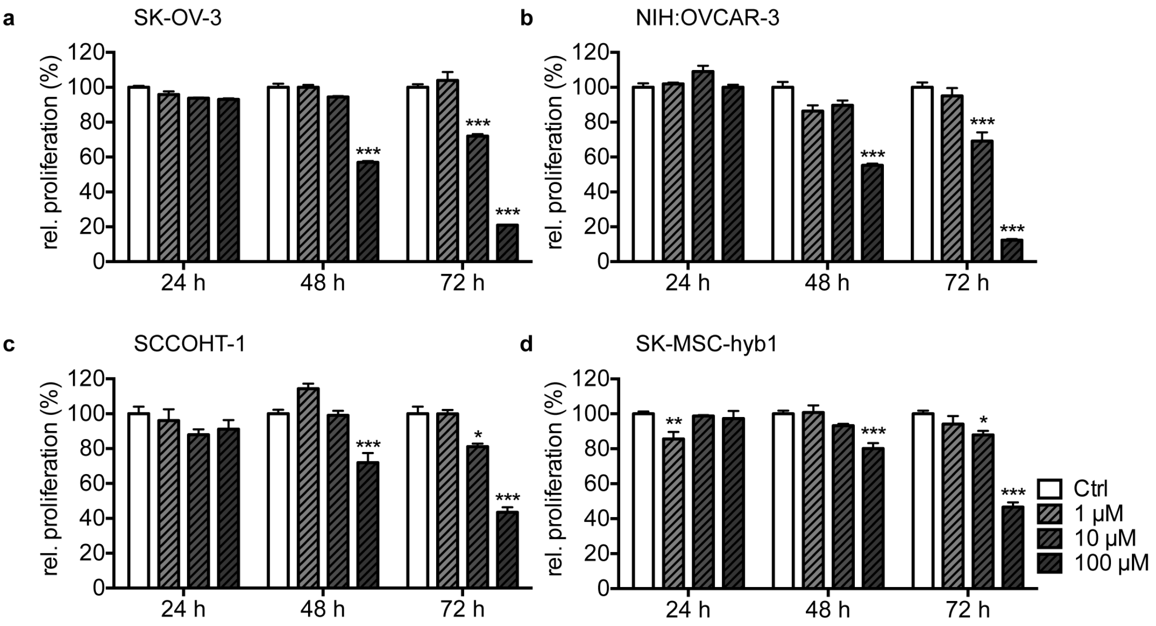

Supplemental Figure S2: Effect of carboplatin treatment on cell proliferation of ovarian cancer cell lines. Bar graphs display relative proliferation rates of SK-OV-3 (**a**), NIH:OVCAR-3 (**b**), SCCOHT-1 (**c**) and SK-MSC-hyb1 (**d**) cells upon exposure to 1 µM, 10 µM or 100 µM carboplatin for indicated time points when compared to DMSO-treated control cells (Ctrl). Data represent results from n = 10 (Crtl) or n = 3 (carboplatin) experiments and are depicted as means ± SEM. *P*-values were obtained by two-way ANOVA followed by Dunnett's multiple comparison test; ****P* < 0.001; ***P* < 0.01; **P* < 0.05 versus corresponding controls.

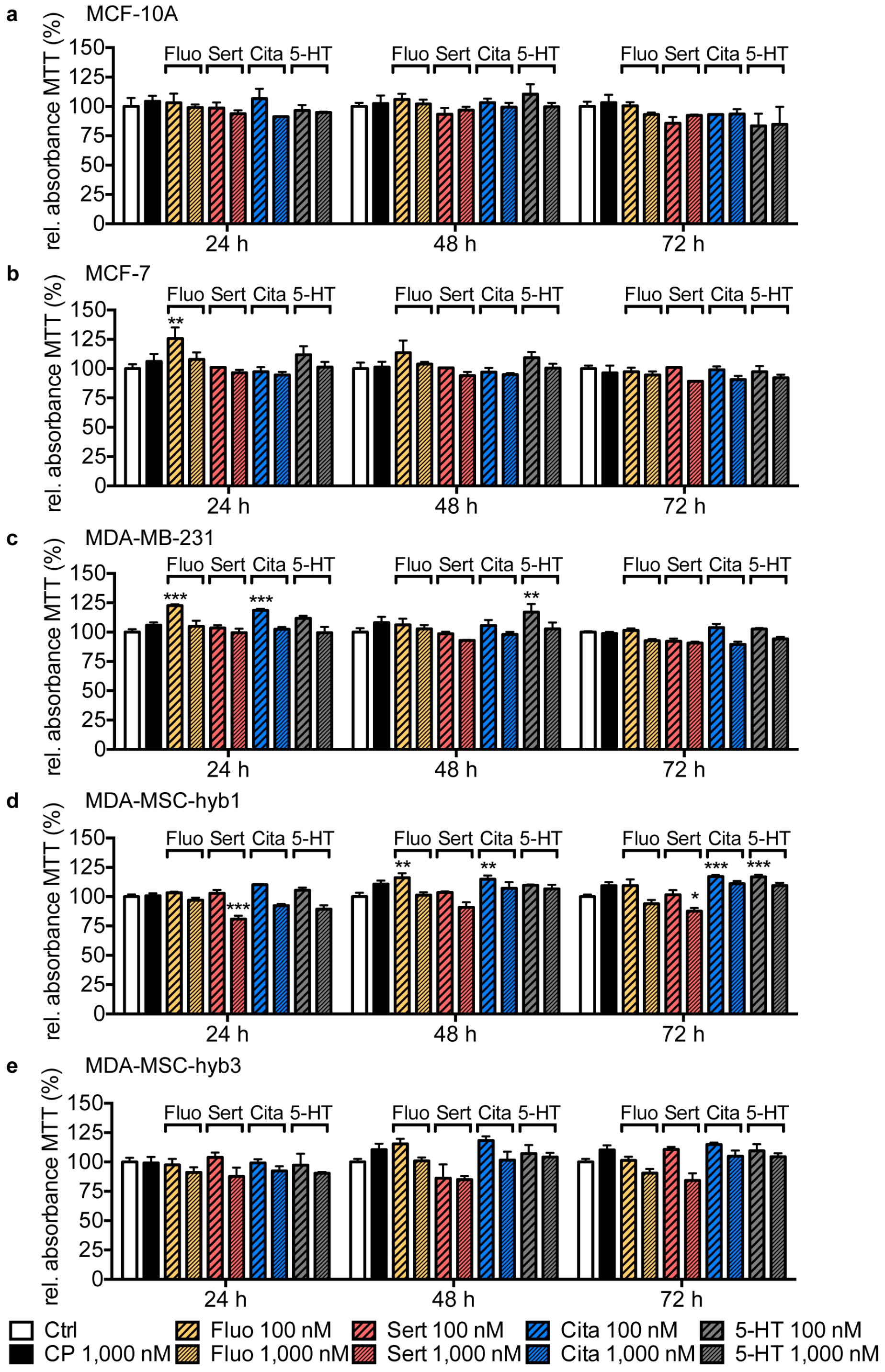

Figure S3: Effect of low-dose, short-term SSRI treatment or 5-HT exposure on breast cancer cell lines measured by MTT assay. Bar graphs depict relative absorbance measured in MTT assay of MCF-10A (**a**), MCF-7 (**b**), MDA-MB-231 (**c**), MDA-MSC-hyb1 (**d**) and MDA-MSC-hyb3 (**e**) cells in response to treatment with 100 nM or 1,000 nM fluoxetine (Fluo; yellow), sertraline (Sert; red), citalopram (Cita; blue) or 5-HT (grey) for indicated time periods (24 h to 72 h) compared to untreated control cells (Ctrl; white) and cells receiving carboplatin (CP; 1,000 nM; black). Data are depicted as mean ± SEM and summarize n = 3 experiments. *P*-values were determined by two-way ANOVA followed by Dunnett's multiple comparison test; ***$P < 0.001$; **$P < 0.01$; *$P < 0.05$ versus corresponding Ctrl.

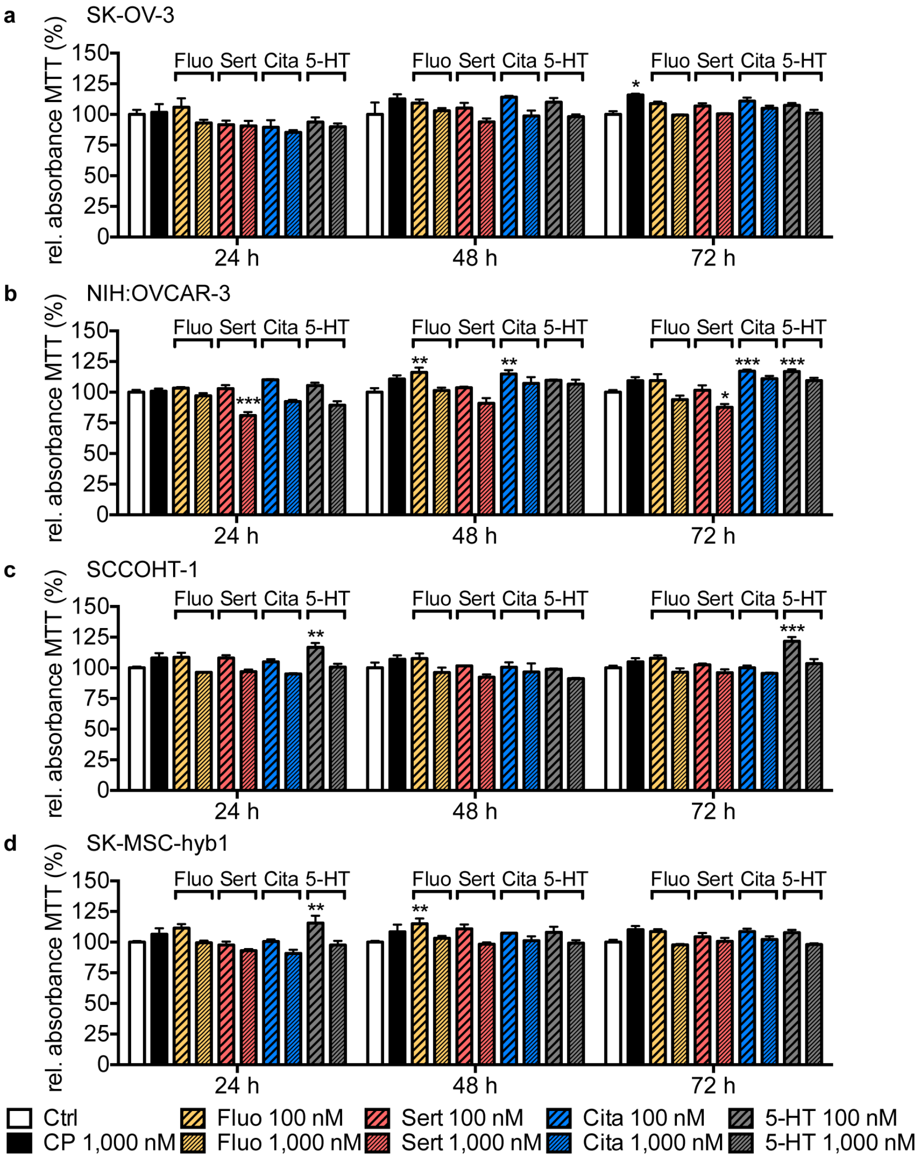

Figure S4: Effect of low-dose, short-term SSRI treatment or 5-HT exposure on ovarian cancer cell lines measured by MTT assay. Bar graphs depict relative absorbance measured in MTT assay of SK-OV-3 (**a**), NIH:OVCAR-3 (**b**), SCCOHT-1 (**c**) and SK-MSC-hyb1 (**d**) cells in response to exposure to 100 nM or 1,000 nM fluoxetine (Fluo; yellow), sertraline (Sert; red), citalopram (Cita; blue) or 5-HT (grey) for indicated time periods (24 h to 72 h) compared to untreated control cells (Ctrl; white) and cell receiving carboplatin (CP; 1,000 nM; black). Data are depicted as mean ± SEM and summarize n = 3 experiments. *P*-values were determined by two-way ANOVA followed by Dunnett's multiple comparison test; ***$P < 0.001$; **$P < 0.01$; *$P < 0.05$ versus corresponding Ctrl.

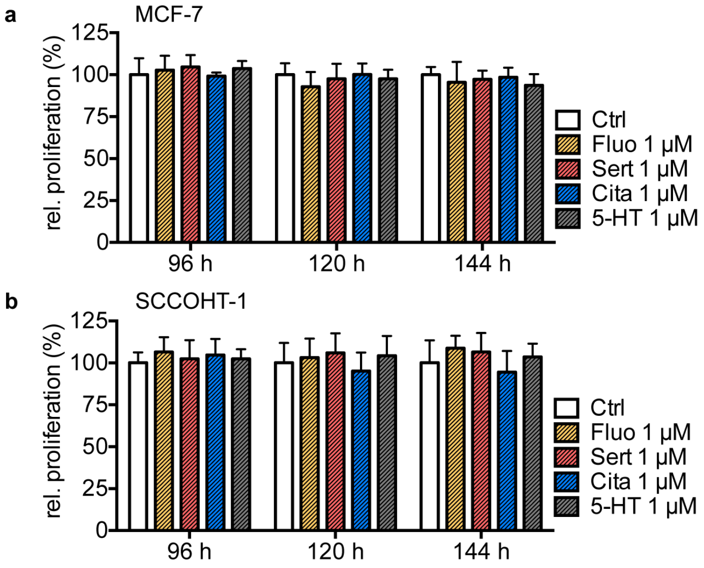

Figure S5: Prolonged stimulation of MCF-7 breast cancer cells or SCCOHT-1 ovarian cancer cells with SSRIs does not affect cell proliferation. Bar graphs depict relative proliferation rate of MCF-7 (**a**) breast cancer cells or SCCOHT-1 (**b**) ovarian cancer cells in response to prolonged (96 h to 144 h) stimulation with 1 µM fluoxetine (Fluo; yellow), sertraline (Sert; red), or citalopram (Cita; blue) in comparison to 5-HT stimulation (1 µM; grey) or corresponding DMSO-treated control cells (Ctrl; white). Data derive from n = 5 experiments and are depicted as means ± SEM. *P*-values were assessed by use of two-way ANOVA followed by Dunnett's multiple comparison test.

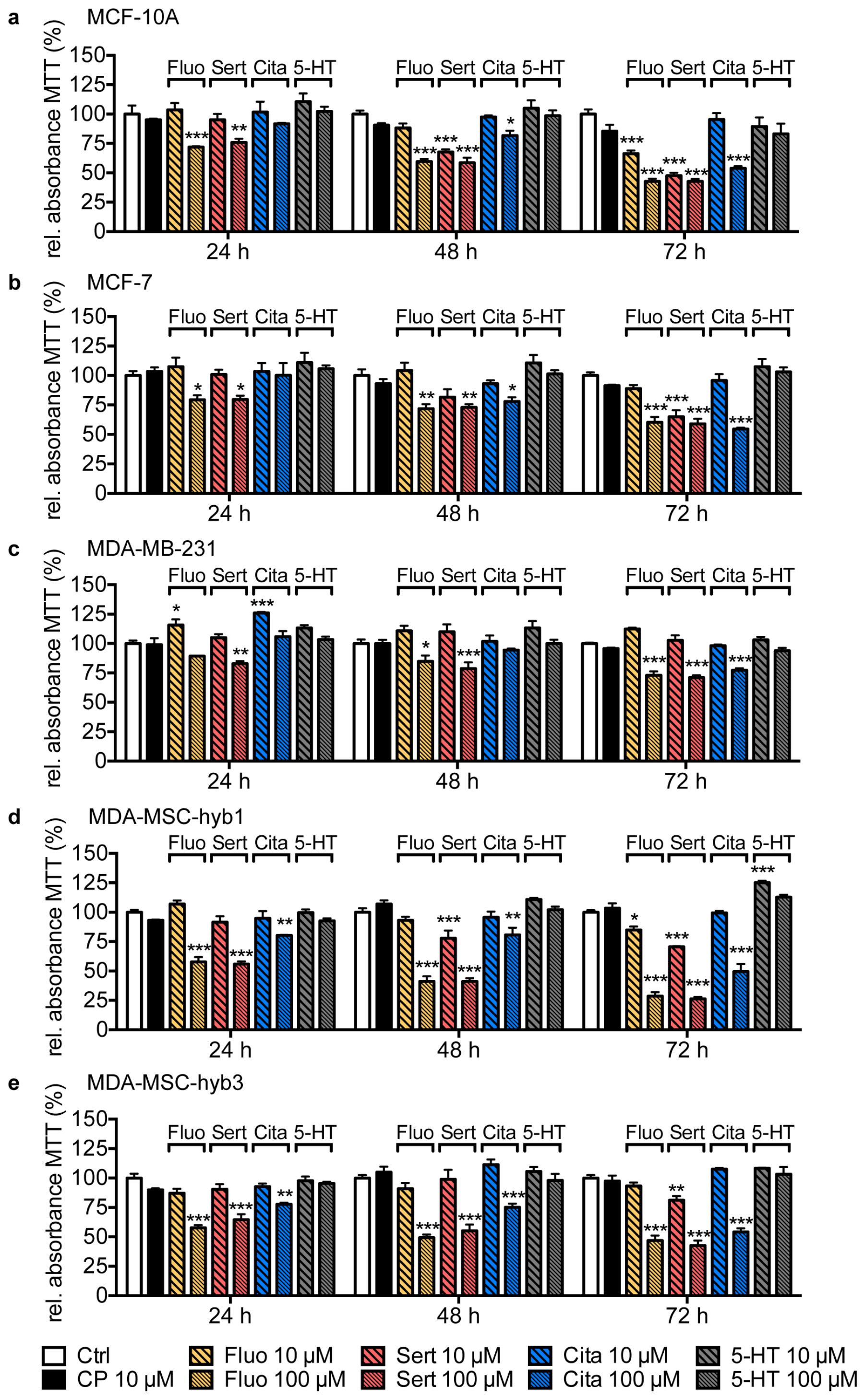

Figure S6: Effect of high-dose, short-term SSRI treatment or 5-HT exposure on breast cancer cell lines measured by MTT assay. Bar graphs depict relative absorbance of MTT assay of MCF-10A (**a**), MCF-7 (**b**), MDA-MB-231 (**c**), MDA-MSC-hyb1 (**d**) and MDA-MSC-hyb3 (**e**) cells in response to treatment with 10 µM or 100 µM fluoxetine (Fluo; yellow), sertraline (Sert; red), citalopram (Cita; blue) or 5-HT (grey) for indicated time periods (24 h to 72 h) compared to untreated control cells (Ctrl; white) and cells receiving carboplatin (CP; 10 µM; black). Data are depicted as mean ± SEM and summarize n = 3 experiments. *P*-values were determined by two-way ANOVA followed by Dunnett's multiple comparison test; ****P* < 0.001; ***P* < 0.01; **P* < 0.05 versus corresponding Ctrl.

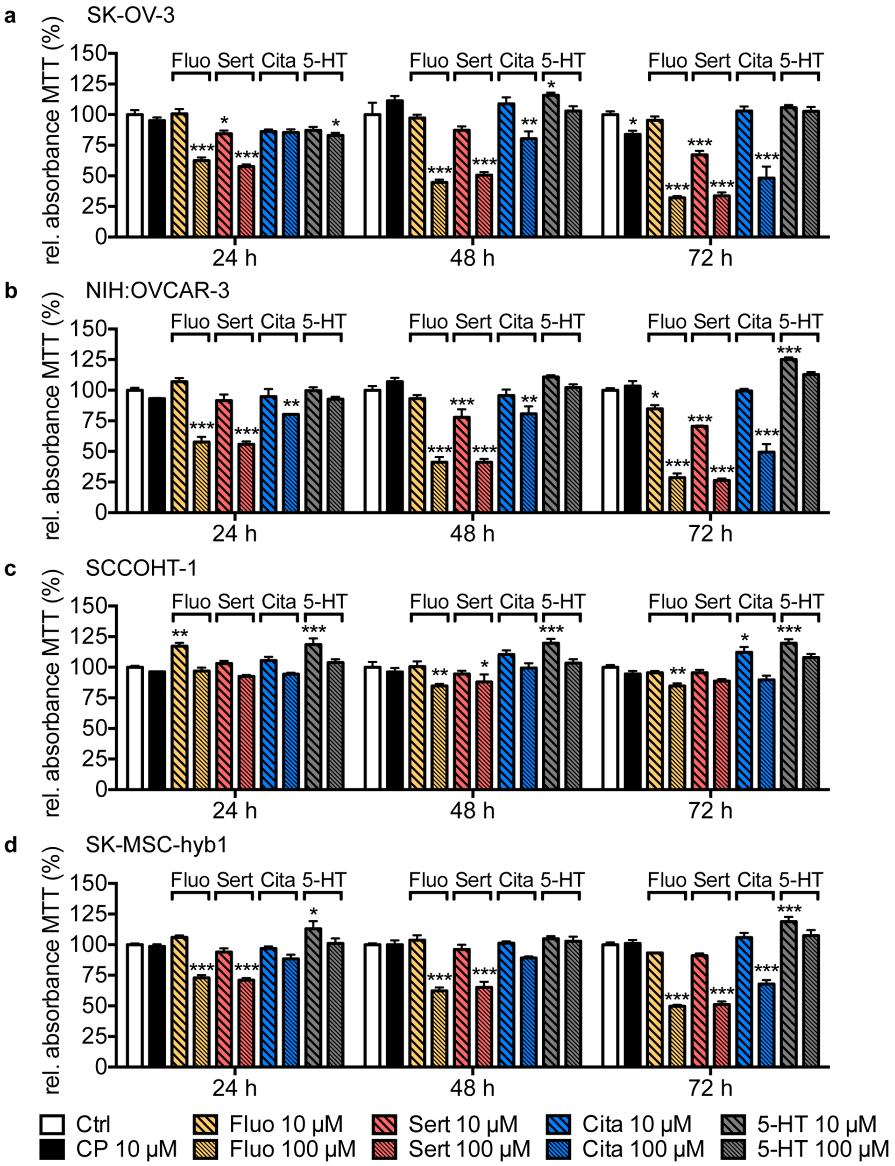

Figure S7: Effect of high-dose, short-term SSRI treatment or 5-HT exposure on ovarian cancer cell lines measured by MTT assay. Bar graphs depict relative absorbance of MTT assay of SK-OV-3 (**a**), NIH:OVCAR-3 (**b**), SCCOHT-1 (**c**) and SK-MSC-hyb1 (**d**) cells in response to exposure to 10 µM or 100 µM fluoxetine (Fluo; yellow), sertraline (Sert; red), citalopram (Cita; blue) or 5-HT (grey) for indicated time periods (24 h to 72 h) compared to untreated control cells (Ctrl; white) and cell receiving carboplatin (CP; 10 µM; black). Data are depicted as mean ± SEM and summarize n = 3 experiments. *P*-values were determined by two-way ANOVA followed by Dunnett's multiple comparison-test; ***$P < 0.001$; **$P < 0.01$; *$P < 0.05$ versus corresponding Ctrl.

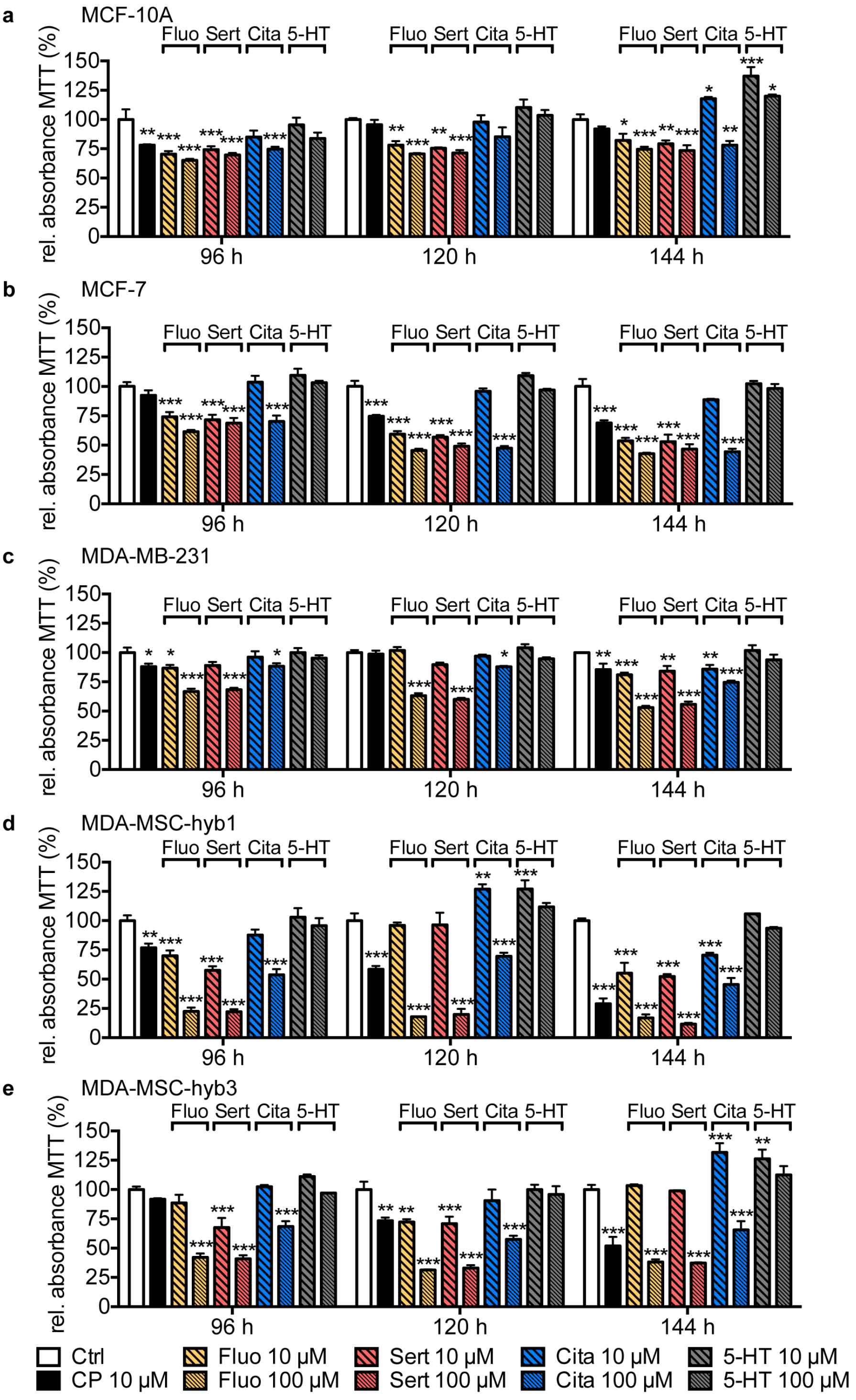

Figure S8: Effect of high-dose, long-term SSRI treatment or 5-HT exposure on breast cancer cell lines measured by MTT assay. Bar graphs show relative absorbance of MTT assay of MCF-10A (**a**), MCF-7 (**b**), MDA-MB-231 (**c**), MDA-MSC-hyb1 (**d**) and MDA-MSC-hyb3 (**e**) cells in response to treatment with 10 µM or 100 µM fluoxetine (Fluo; yellow), sertraline (Sert; red), citalopram (Cita; blue) or 5-HT (grey) for indicated time periods (96 h to 144 h) compared to DMSO-treated control cells (Ctrl; white) and cells receiving carboplatin (CP; 10 µM; black). Data are depicted as mean ± SEM and summarize n = 3 experiments. *P*-values were determined by two-way ANOVA followed by Dunnett's multiple comparison test; ***$P < 0.001$; **$P < 0.01$; *$P < 0.05$ versus corresponding Ctrl.

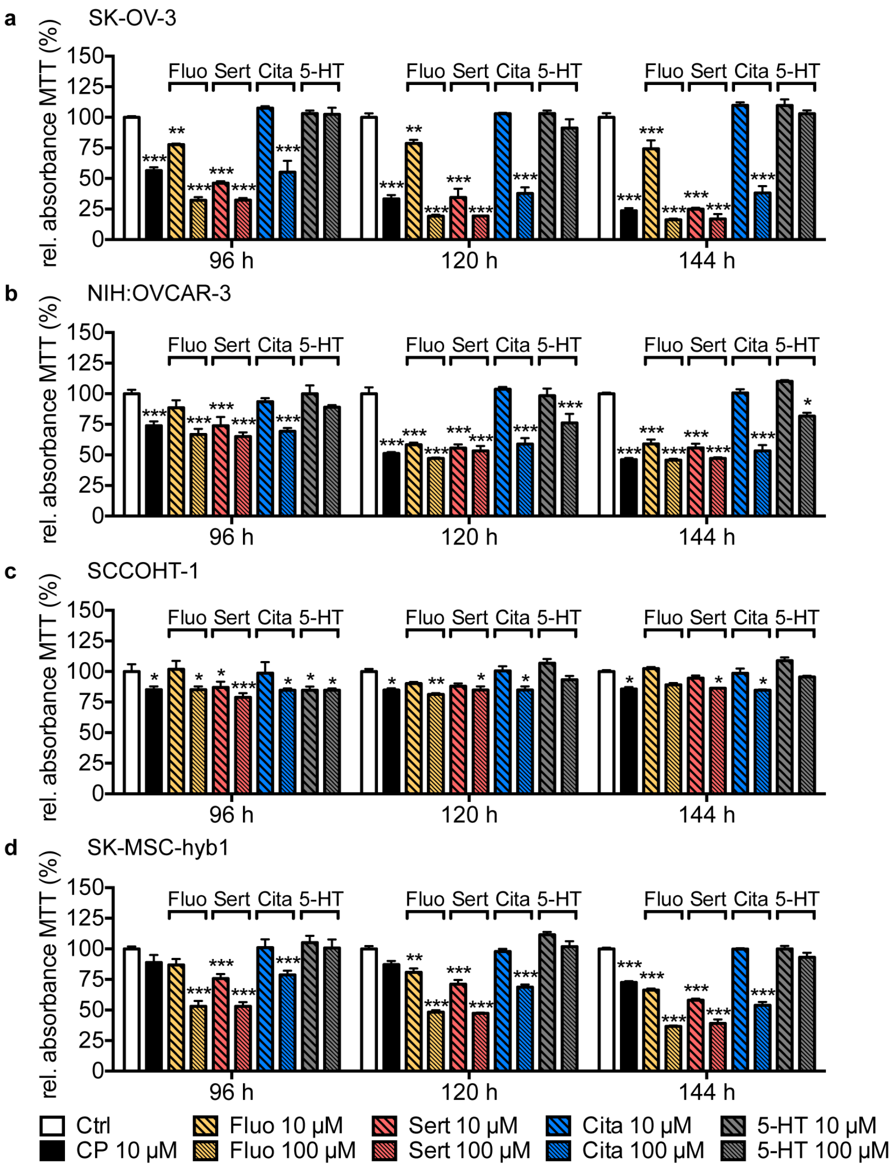

Figure S9: Effect of high-dose, long-term SSRI treatment or 5-HT exposure on ovarian cancer cell lines measured by MTT assay. Bar graphs depict relative absorbance measured by MTT assay of SK-OV-3 (**a**), NIH:OVCAR-3 (**b**), SCCOHT-1 (**c**) and SK-MSC-hyb1 (**d**) cells in response to exposure to 10 µM or 100 µM fluoxetine (Fluo; yellow), sertraline (Sert; red), citalopram (Cita; blue) or 5-HT (grey) for indicated time periods (96 h to 144 h) compared to DMSO-treated control cells (Ctrl; white) and cell receiving carboplatin (CP; 10 µM; black). Data are depicted as mean ± SEM and summarize n = 3 experiments. *P*-values were determined by two-way ANOVA followed by Dunnett's multiple comparison-test; ****P* < 0.001; ***P* < 0.01; **P* < 0.05 versus corresponding Ctrl.

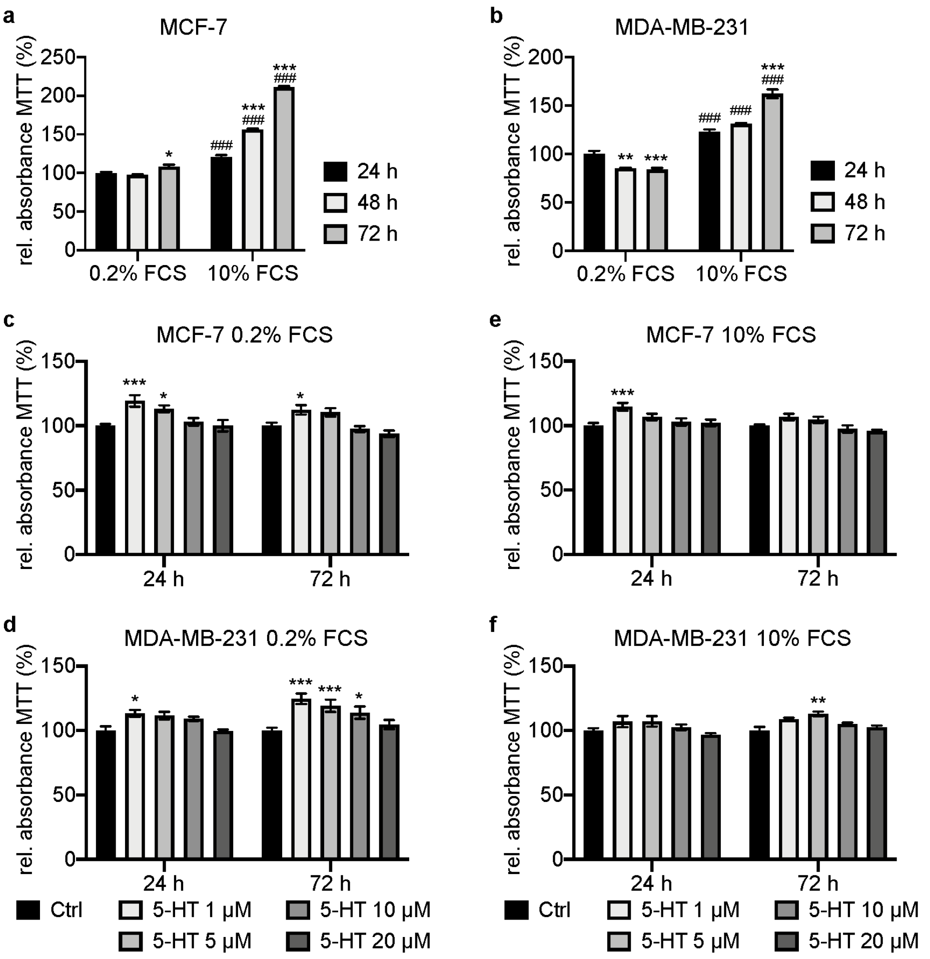

Figure S10: FCS concentration impacts 5-HT-mediated effect on cell count in MCF-7 and MDA-MB-231 breast cancer cells. Bar graphs show relative absorbance of MTT in indicated breast cancer cell lines. Effects of different FCS concentrations in the culture medium are depicted in **a** and **b**. The effect of 5-HT stimulation (1 µM – 20 µM) for 24 h or 72 h in the presences of 0.2% FCS is shown for indicated cell lines in **c** and **d**, while effects of 5-HT in the presences of 10% FCS are depicted in **e** and **f**. Data are visualized as mean ± SEM and summarize n = 4-5 experiments. Two-way ANOVA followed by either Dunnett's multiple comparison test was used to calculate two-tailed *P*-values to compare 5-HT groups to respective Ctrl, or by Sidak's multiple comparison test to assess differences between corresponding time points (a and b). ***$P < 0.001$, **$P < 0.01$, *$P < 0.05$ versus corresponding Ctrl, ###$P < 0.001$ versus corresponding time point with 0.2% FCS.

Supplemental Table S2: Summary of Figure 1 detailing time-points and drug concentrations resulting in statistically significant changes in relative proliferation rate of indicated breast cancer cell lines when compared to DMSO-treated control cells.

		Fluoxetine	Sertraline	Citalopram	5-HT
breast cancer cell lines	MCF-10A	t 24, c 1,000, *P = 0.0461*		t 24, c 100, *P = 0.0172*	
	MCF-7	**t 72, c 100, *P = 0.0423***		t 24, c 1,000, *P = 0.0119*	
	MDA-MB-231	t 24, c 1,000, *P < 0.0001*<br>t 48, c 1,000, *P = 0.0104*<br>t 72, c 1,000, *P < 0.0001*	t 48, c 100, *P = 0.0010*	t 48, c 1,000, *P = 0.0113*	t 72, c 100, *P = 0.0354*
	MDA-MSC-hyb1	t 24, c 10, *P = 0.0018*<br>t 24, c 100, *P < 0.0001*<br>t 24, c 1,000, *P = 0.0034*	t 24, c 10, *P = 0.0028*<br>t 24, c 100, *P = 0.0019*<br>t 24, c 1,000, *P = 0.0002*<br>t 72, c 10, *P = 0.0190*<br>t 72, c 100, *P = 0.0001*<br>t 72, c 1,000, *P = 0.0006*	t 24, c 100, *P < 0.0001*<br>t 24, c 1,000, *P < 0.0001*<br>t 48, c 100, *P = 0.0264*<br>t 48, c 1,000, *P = 0.0080*	**t 72, c 10, *P = 0.0019***
	MDA-MSC-hyb3	t 48, c 10, *P = 0.0125*	t 72, c 100, *P = 0.0049*		

c: drug concentration in nM, t: time-point in hours. *P*-values were determined by two-way ANOVA followed by Dunnett's multiple comparison test. Conditions resulting in a significant up-regulation of proliferation rates are indicated in bold print.

Supplemental Table S3: Summary of Figure 2 detailing time-points and drug concentrations resulting in statistically significant changes in relative proliferation rate of indicated ovarian cancer cell lines when compared to DMSO-treated control cells.

		Fluoxetine	Sertraline	Citalopram	5-HT
ovarian cancer cell lines	SK-OV-3				
	NIH:OVCAR-3				
	SCCOHT-1				
	SK-MSC-hyb1	t 24, c 1,000, *P = 0.0366*	t 48, c 100, *P = 0.0201*<br>t 72, c 100, *P = 0.0125*	t 48, c 10, *P = 0.0015*<br>t 48, c 100, *P = 0.0324*	

c: drug concentration in nM, t: time-point in hours. *P*-values were determined by two-way ANOVA followed by Dunnett's multiple comparison test. Conditions resulting in a significant up-regulation of proliferation rates are indicated in bold print.

Supplemental Table S4: Summary of Figure S3 detailing time-points and drug concentrations resulting in statistically significant changes in relative proliferation rate of indicated breast cancer cell lines when compared to untreated control cells.

		Fluoxetine	Sertraline	Citalopram	5-HT	Carboplatin
breast cancer cell lines	MCF-10A					
	MCF-7	**t 24, c 100, *P = 0.0014***				
	MDA-MB-231	**t 24, c 100, *P < 0.0001***		**t 24, c 100, *P = 0.0009***	**t 48, c 100, *P = 0.0030***	
	MDA-MSC-hyb1	**t 48, c 100, *P = 0.0013***	t 24, c 1,000, *P = 0.0001*<br>t 72, c 1,000, *P = 0.0244*	**t 48, c 100, *P = 0.0037***<br>**t 72, c 100, *P = 0.0006***	**t 72, c 100, *P = 0.0008***	
	MDA-MSC-hyb3					

c: drug concentration in nM, t: time-point in hours. *P*-values were determined by two-way ANOVA followed by Dunnett's multiple comparison test. Conditions resulting in a significant up-regulation of proliferation rates are indicated in bold print.

Supplemental Table S5: Summary of Figure S4 detailing time-points and drug concentrations resulting in statistically significant changes in relative proliferation rate of indicated ovarian cancer cell lines when compared to untreated control cells.

		Fluoxetine	Sertraline	Citalopram	5-HT	Carboplatin
ovarian cancer cell lines	SK-OV-3					**t 72, c 1,000, *P = 0.0315***
	NIH:OVCAR-3	t 48, c 100, *P = 0.0013*	t 24, c 1,000, *P = 0.0001*<br>t 72, c 1,000, *P = 0.0244*	**t 48, c 100, *P = 0.0037***<br>**t 72, c 100, *P = 0.0006***	**t 72, c 100, *P = 0.0008***	
	SCCOHT-1				**t 24, c 100, *P = 0.0015***<br>**t72, c 100, *P < 0.0001***	
	SK-MSC-hyb1	**t 48, c 100, *P = 0.0063***			**t 24, c 100, *P = 0.0038***	

c: drug concentration in nM, t: time-point in hours. *P*-values were determined by two-way ANOVA followed by Dunnett's multiple comparison test. Conditions resulting in a significant up-regulation of proliferation rates are indicated in bold print.

Supplemental Table S6: Summary of Figure 5 detailing time-points and drug concentrations resulting in statistically significant changes in relative proliferation rate of indicated breast cancer cell lines when compared to untreated control cells.

		Fluoxetine	Sertraline	Citalopram	5-HT	Carboplatin
breast cancer cell lines	MCF-10A	t 96, c 1,000, *P = 0.0052*<br>**t 144, c 100, *P = 0.0033***	t 96, c 1,000, *P = 0.0165*<br>**t 144, c 100, *P = 0.0391***<br>**t 144, c 1,000, *P = 0.0076***	t 96, c 100, *P = 0.0165*<br>t 96, c 1,000, *P = 0.0052*<br>**t 144, c 100, *P = 0.0024***<br>**t 144, c 1,000, *P = 0.0449***	t 96, c 100, *P = 0.0193*<br>t 96, c 1,000, *P = 0.0353*<br>**t 144, c 100, *P = 0.0040***	
	MCF-7	**t 96, c 100, *P = 0.0045***<br>**t 96, c 1,000, *P = 0.0290***	t 120, c 1,000, *P = 0.0082*			
	MDA-MB-231	**t 120, c 100, *P < 0.0001***<br>t 144, c 100, *P = 0.0266*<br>t 144, c 1,000, *P = 0.0005*	t 144, c 1,000, *P = 0.0077*	t 96, c 100, *P = 0.0201*<br>t 96, c 1,000, *P = 0.0058*<br>t 144, c 1,000, *P = 0.0062*		
	MDA-MSC-hyb1	**t 120, c 1,000, *P = 0.0017***<br>t 144, c 100, *P < 0.0001*<br>t 144, c 1,000, *P < 0.0001*	t 96, c 1,000, *P = 0.0004*<br>t 144, c 1,000, *P = 0.0038*	**t 120, c 100, *P = 0.0102***		
	MDA-MSC-hyb3	t 120, c 1,000, *P = 0.0397*<br>**t 144, c 100, *P = 0.0012***	**t 144, c 100, *P = 0.0005***	**t 144, c 100, *P < 0.0001***<br>**t 144, c 1,000, *P = 0.0109***	**t 144, c 100, *P = 0.0219***	

c: drug concentration in nM, t: time-point in hours. *P*-values were determined by two-way ANOVA followed by Dunnett's multiple comparison test. Conditions resulting in a significant up-regulation of proliferation rates are indicated in bold print.

Supplemental Table S7: Summary of Figure 5 detailing time-points and drug concentrations resulting in statistically significant changes in relative proliferation rate of indicated ovarian cancer cell lines when compared to untreated control cells.

		Fluoxetine	Sertraline	Citalopram	5-HT	Carboplatin
ovarian cancer cell lines	SK-OV-3					
	NIH:OVCAR-3	t 120, c 1,000, *P = 0.0050*		t 120, c 100, *P = 0.0069*<br>t 120, c 1,000, *P = 0.0096*<br>t 144, c 1,000, *P = 0.0361*		t 120, c 100, *P = 0.0011*<br>t 144, c 100, *P = 0.0006*
	SCCOHT-1	t 96, c 1,000, *P = 0.0274*<br>t 120, c 1,000, *P = 0.0211*	t 96, c 100, *P = 0.0109*<br>t 96, c 1,000, *P = 0.0005*<br>t 120, c 1,000, *P = 0.0032*<br>t 144, c 1,000, *P = 0.0451*	t 96, c 1,000, *P = 0.0485*<br>t 120, c 100, *P = 0.0416*<br>t 120, c 1,000, *P = 0.0047*<br>t 144, c 1,000, *P = 0.0329*	t 96, c 100, *P = 0.0109*<br>t 96, c 1,000, *P = 0.0003*	
	SK-MSC-hyb1	t 144, c 1,000, *P = 0.0176*	t 144, c 1,000, *P = 0.0036*			

c: drug concentration in nM, t: time-point in hours. *P*-values were determined by two-way ANOVA followed by Dunnett's multiple comparison test. Conditions resulting in a significant up-regulation of proliferation rates are indicated in bold print.

Supplemental Table S8: Summary of Figure S6 detailing time-points and drug concentrations resulting in statistically significant changes in relative proliferation rate of indicated breast cancer cell lines when compared to DMSO-treated control cells.

		Fluoxetine	Sertraline	Citalopram	5-HT	Carboplatin
breast cancer cell lines	MCF-10A	t 24, c 100, *P = 0.0006*<br>t 48, c 100, *P < 0.0001*<br>t 72, c 10, *P < 0.0001*<br>t 72, c 100, *P < 0.0001*	t 24, c 100, *P = 0.0038*<br>t 48, c 10, *P < 0.0001*<br>t 48, c 100, *P < 0.0001*<br>t 72, c 10, *P < 0.0001*<br>t 72, c 100, *P < 0.0001*	t 48, c 100, *P = 00473*<br>t 72, c 100, *P < 0.0001*		
	MCF-7	t 24, c 100, *P = 0.0354*<br>t 48, c 100, *P = 0.0017*<br>t 72, c 100, *P < 0.0001*	t 24, c 100, *P = 0.0415*<br>t 48, c 100, *P = 0.0031*<br>t 72, c 10, *P < 0.0001*<br>t 72, c 100, *P < 0.0001*	48, c 100, *P = 0.0209*<br>72, c 100, *P < 0.0001*		
	MDA-MB-231	**t 24, c 10, *P = 0.0176***<br>t 48, c 100, *P = 0.0244*<br>t 72, c 100, *P < 0.0001*	t 24, c 100, *P = 0.0075*<br>t 48, c 100, *P = 0.0006*<br>t 72, c 100, *P < 0.0001*	**t 24, c 10, *P < 0.0001***<br>t 72, c 100, *P = 0.0002*		
	MDA-MSC-hyb1	t 24, c 100, *P < 0.0001*<br>t 48, c 100, *P < 0.0001*<br>t 72, c 10, *P = 0.0258*<br>t 72, c 100, *P < 0.0001*	t 24, c 100, *P < 0.0001*<br>t 48, c 10, *P = 0.0004*<br>t 48, c 100, *P < 0.0001*<br>t 72, c 10, *P < 0.0001*<br>t 72, c 100, *P < 0.0001*	t 24, c 100, *P = 0.0017*<br>t 48, c 100, *P = 0.0022*<br>t 72, c 100, *P < 0.0001*	**t 72, c 10, *P < 0.0001***	
	MDA-MSC-hyb3	t 24, c 100, *P < 0.0001*<br>t 48, c 100, *P < 0.0001*<br>t 72, c 100, *P < 0.0001*	t 24, c 100, *P < 0.0001*<br>t 48, c 100, *P < 0.0001*<br>t 72, c 10, *P = 0.0090*<br>t 72, c 100, *P < 0.0001*	t 24, c 100, *P = 0.0013*<br>t 48, c 100, *P = 0.0003*<br>t 72, c 100, *P < 0.0001*		

c: drug concentration in µM, t: time-point in hours. *P*-values were determined by two-way ANOVA followed by Dunnett's multiple comparison test. Conditions resulting in a significant up-regulation of proliferation rates are indicated in bold print.

Supplemental Table S9: Summary of Figure S7 detailing time-points and drug concentrations resulting in statistically significant changes in relative proliferation rate of indicated ovarian cancer cell lines when compared to DMSO-treated control cells.

		Fluoxetine	Sertraline	Citalopram	5-HT	Carboplatin
ovarian cancer cell lines	SK-OV-3	t 24, c 100, *P < 0.0001*<br>t 48, c 100, *P < 0.0001*<br>t 72, c 100, *P < 0.0001*	t 24, c 10, *P = 0.0447*<br>t 24, c 100, *P < 0.0001*<br>t 48, c 100, *P < 0.0001*<br>t 72, c 10, *P < 0.0001*<br>t 72, c 100, *P < 0.0001*	t 48, c 100, *P = 0.0059*<br>t 72, c 100, *P < 0.0001*	t 24, c 100, *P = 0.0233*<br>**t 48, c 10, *P = 0.0421***	t 72, c 10, *P = 0.0346*
	NIH:OVCAR-3	t 24, c 100, *P < 0.0001*<br>t 48, c 100, *P < 0.0001*<br>t 72, c 10, *P = 0.0258*<br>t 72, c 100, *P < 0.0001*	t 24, c 100, *P < 0.0001*<br>t 48, c 10, *P = 0.0004*<br>t 48, c 100, *P < 0.0001*<br>t 72, c 10, *P < 0.0001*<br>t 72, c 100, *P < 0.0001*	t 24, c 100, *P = 0.0017*<br>t 48, c 100, *P = 0.0022*<br>t 72, c 100, *P < 0.0001*	**t 72, c 10, *P < 0.0001***	
	SCCOHT-1	**t 24, c 10, *P = 0.0014***<br>t 48, c 100, *P = 0.0060*<br>t 72, c 100, *P = 0.0061*	t 48, c 100, *P = 0.0484*	**t 72, c 10, *P = 0.0409***	**t 24, c 10, *P = 0.0006***<br>**t 48, c 10, *P = 0.0002***<br>**t 72, c 10, *P = 0.0002***	
	SK-MSC-hyb1	t 24, c 100, *P < 0.0001*<br>t 48, c 100, *P < 0.0001*<br>t 72, c 100, *P < 0.0001*	t 24, c 100, *P < 0.0001*<br>t 48, c 100, *P < 0.0001*<br>t 72, c 100, *P < 0.0001*	t 72, c 100, *P < 0.0001*	**t 24, c 10, *P = 0.0262***<br>**t 72, c 10, *P = 0.0004***	

c: drug concentration in µM, t: time-point in hours. *P*-values were determined by two-way ANOVA followed by Dunnett's multiple comparison test. Conditions resulting in a significant up-regulation of proliferation rates are indicated in bold print.

Supplemental Table S10: Summary of Figure S8 detailing time-points and drug concentrations resulting in statistically significant changes in relative proliferation rate of indicated breast cancer cell lines when compared to untreated control cells.

		Fluoxetine	Sertraline	Citalopram	5-HT	Carboplatin
breast cancer cell lines	MCF-10A	t 96, c 10, *P < 0.0001*<br>t 96, c 100, *P < 0.0001*<br>t 120, c 10, *P = 0.0054*<br>t 120, c 100, *P < 0.0001*<br>t 144, c 10, *P = 0.0349*<br>t 144, c 100, *P = 0.0009*	t 96, c 10, *P = 0.0008*<br>t 96, c 100, *P < 0.0001*<br>t 120, c 10, *P = 0.0014*<br>t 120, c 100, *P = 0.0002*<br>t 144, c 10, *P = 0.0095*<br>t 144, c 100, *P = 0.0005*	t 96, c 100, *P = 0.0009*<br>t 144, c 10, *P = 0.0349*<br>t 144, c 100, *P = 0.0056*	**t 144, c 10, *P < 0.0001***<br>**t 144, c 100, *P = 0.0133***	t 96, c 10, *P = 0.0055*
	MCF-7	t 96, c 10, *P < 0.0001*<br>t 96, c 100, *P < 0.0001*<br>t 120, c 10, *P < 0.0001*<br>t 120, c 100, *P < 0.0001*<br>t 144, c 10, *P < 0.0001*<br>t 144, c 100, *P < 0.0001*	t 96, c 10, *P < 0.0001*<br>t 96, c 100, *P < 0.0001*<br>t 120, c 10, *P < 0.0001*<br>t 120, c 100, *P < 0.0001*<br>t 144, c 10, *P < 0.0001*<br>t 144, c 100, *P < 0.0001*	t 96, c 100, *P < 0.0001*<br>t 120, c 100, *P < 0.0001*<br>t 144, c 100, *P < 0.0001*		t 120, c 10, *P < 0.0001*<br>t 144, c 10, *P < 0.0001*
	MDA-MB-231	t 96, c 10, *P = 0.0156*<br>t 96, c 100, *P < 0.0001*<br>t 120, c 100, *P < 0.0001*<br>t 144, c 10, *P = 0.0002*<br>t 144, c 100, *P < 0.0001*	t 96, c 100, *P < 0.0001*<br>t 120, c 100, *P < 0.0001*<br>t 144, c 10, *P = 0.0026*<br>t 144, c 100, *P < 0.0001*	t 96, c 100, *P = 0.0426*<br>t 120, c 100, *P = 0.0332*<br>t 144, c 10, *P = 0.0085*<br>t 144, c 100, *P < 0.0001*		t 96, c 10, *P = 0.0334*<br>t 144, c 10, *P = 0.0058*
	MDA-MSC-hyb1	t 96, c 10, *P = 0.0002*<br>t 96, c 100, *P < 0.0001*<br>t 120, c 100, *P < 0.0001*<br>t 144, c 10, *P < 0.0001*<br>t 144, c 100, *P < 0.0001*	t 96, c 10, *P < 0.0001*<br>t 96, c 100, *P < 0.0001*<br>t 120, c 100, *P < 0.0001*<br>t 144, c 10, *P < 0.0001*<br>t 144, c 100, *P < 0.0001*	t 96, c 100, *P < 0.0001*<br>**t 120, c 10, *P = 0.0011***<br>t 120, c 100, *P = 0.0002*<br>t 144, c 10, *P = 0.0003*<br>t 144, c 100, *P < 0.0001*	**t 120, c 10, *P = 0.0010***	t 96, c 10, *P = 0.0067*<br>t 120, c 10, *P < 0.0001*<br>t 144, c 10, *P < 0.0001*
	MDA-MSC-hyb3	t 96, c 100, *P < 0.0001*<br>t 120, c 10, *P = 0.0018*<br>t 120, c 100, *P < 0.0001*<br>t 144, c 100, *P < 0.0001*	t 96, c 10, *P = 0.0002*<br>t 96, c 100, *P < 0.0001*<br>t 120, c 10, *P = 0.0010*<br>t 120, c 100, *P < 0.0001*<br>t 144, c 100, *P < 0.0001*	t 96, c 100, *P = 0.0003*<br>t 120, c 100, *P < 0.0001*<br>**t 144, c 10, *P = 0.0003***<br>t 144, c 100, *P < 0.0001*	**t 144, c 10, *P = 0.0037*** **	t 120, c 10, *P = 0.0031*<br>t 144, c 10, *P < 0.0001*

c: drug concentration in µM, t: time-point in hours. *P*-values were determined by two-way ANOVA followed by Dunnett's multiple comparison test. Conditions resulting in a significant up-regulation of proliferation rates are indicated in bold print.

Supplemental Table S11: Summary of Figure S9 detailing time-points and drug concentrations resulting in statistically significant changes in relative proliferation rate of indicated ovarian cancer cell lines when compared to untreated control cells.

		Fluoxetine	Sertraline	Citalopram	5-HT	Carboplatin
ovarian cancer cell lines	SK-OV-3	t 96, c 10, $P = 0.0014$<br>t 96, c 100, $P < 0.0001$<br>t 120, c 10, $P = 0.0024$<br>t 120, c 100, $P < 0.0001$<br>t 144, c 10, $P = 0.0002$<br>t 144, c 100, $P < 0.0001$	t 96, c 10, $P < 0.0001$<br>t 96, c 100, $P < 0.0001$<br>t 120, c 10, $P < 0.0001$<br>t 120, c 100, $P < 0.0001$<br>t 144, c 10, $P < 0.0001$<br>t 144, c 100, $P < 0.0001$	t 96, c 100, $P < 0.0001$<br>t 120, c 100, $P < 0.0001$<br>t 144, c 100, $P < 0.0001$		t 96, c 10, $P < 0.0001$<br>t 120, c 10, $P < 0.0001$<br>t 144, c 10, $P < 0.0001$
	NIH:OVCAR-3	t 96, c 100, $P < 0.0001$<br>t 120, c 10, $P < 0.0001$<br>t 120, c 100, $P < 0.0001$<br>t 144, c 10, $P < 0.0001$<br>t 144, c 100, $P < 0.0001$	t 96, c 10, $P = 0.0001$<br>t 96, c 100, $P < 0.0001$<br>t 120, c 10, $P < 0.0001$<br>t 120, c 100, $P < 0.0001$<br>t 144, c 10, $P < 0.0001$<br>t 144, c 100, $P < 0.0001$	t 96, c 100, $P < 0.0001$<br>t 120, c 100, $P < 0.0001$<br>t 144, c 100, $P < 0.0001$	t 120, c 100, $P = 0.0004$<br>t 144, c 100, $P = 0.0107$	t 96, c 10, $P = 0.0001$<br>t 120, c 10, $P < 0.0001$<br>t 144, c 10, $P < 0.0001$
	SCCOHT-1	t 96, c 100, $P = 0.0180$<br>t 120, c 100, $P = 0.0014$	t 96, c 10, $P = 0.0493$<br>t 96, c 100, $P = 0.0003$<br>t 120, c 100, $P = 0.0151$<br>t 144, c 100, $P = 0.0321$	t 96, c 100, $P = 0.0138$<br>t 120, c 100, $P = 0.0151$<br>t 144, c 100, $P = 0.0136$	t 96, c 10, $P = 0.0138$<br>t 96, c 100, $P = 0.0138$	t 96, c 10, $P = 0.0180$*<br>t 120, c 10, $P = 0.0151$<br>t 144, c 10, $P = 0.0243$
	SK-MCS-hyb1	t 96, c 100, $P < 0.0001$<br>t 120, c 10, $P = 0.0021$<br>t 120, c 100, $P < 0.0001$<br>t 144, c 10, $P < 0.0001$<br>t 144, c 100, $P < 0.0001$	t 96, c 10, $P < 0.0001$<br>t 96, c 100, $P < 0.0001$<br>t 120, c 10, $P < 0.0001$<br>t 120, c 100, $P < 0.0001$<br>t 144, c 10, $P < 0.0001$<br>t 144, c 100, $P < 0.0001$	t 96, c 100, $P = 0.0005$<br>t 120, c 100, $P < 0.0001$<br>t 144, c 100, $P < 0.0001$		t 144, c 10, $P < 0.0001$

c: drug concentration in µM, t: time-point in hours. *P*-values were determined by two-way ANOVA followed by Dunnett's multiple comparison test. Conditions resulting in a significant up-regulation of proliferation rates are indicated in bold print.

Supplemental References

1. Soule, H.D. *et al.* Isolation and characterization of a spontaneously immortalized human breast epithelial cell line, MCF-10. *Cancer research* **50**, 6075-6086 (1990).
2. Soule, H.D., Vazguez, J., Long, A., Albert, S. & Brennan, M. A human cell line from a pleural effusion derived from a breast carcinoma. *Journal of the National Cancer Institute* **51**, 1409-1416 (1973).
3. Cailleau, R., Olive, M. & Cruciger, Q.V. Long-term human breast carcinoma cell lines of metastatic origin: preliminary characterization. *In vitro* **14**, 911-915 (1978).
4. Melzer, C., von der Ohe, J. & Hass, R. Enhanced metastatic capacity of breast cancer cells after interaction and hybrid formation with mesenchymal stroma/stem cells (MSC). *Cell communication and signaling : CCS* **16**, 2 (2018).
5. Melzer, C., von der Ohe, J. & Hass, R. In Vivo Cell Fusion between Mesenchymal Stroma/Stem-Like Cells and Breast Cancer Cells. *Cancers* **11** (2019).
6. Fogh, J. & Trempe, G. New Human Tumor Cell Lines. In: Fogh J. (eds) Human Tumor Cells in Vitro. Springer, Boston, MA (1975).
7. Hamilton, T.C. *et al.* Characterization of a human ovarian carcinoma cell line (NIH:OVCAR-3) with androgen and estrogen receptors. *Cancer research* **43**, 5379-5389 (1983).
8. Otte, A. *et al.* In vitro and in vivo therapeutic approach for a small cell carcinoma of the ovary hypercalcaemic type using a SCCOHT-1 cellular model. *Orphanet journal of rare diseases* **9**, 126 (2014).
9. Otte, A. *et al.* A tumor-derived population (SCCOHT-1) as cellular model for a small cell ovarian carcinoma of the hypercalcemic type. *International journal of oncology* **41**, 765-775 (2012).
10. Melzer, C., von der Ohe, J. & Hass, R. In Vitro Fusion of Normal and Neoplastic Breast Epithelial Cells with Human Mesenchymal Stroma/Stem Cells Partially Involves Tumor Necrosis Factor Receptor Signaling. *Stem cells* **36**, 977-989 (2018).
